# Differential Protein Modulation in Midguts of *Aedes aegypti* Infected with Chikungunya and Dengue 2 Viruses

**DOI:** 10.1371/journal.pone.0013149

**Published:** 2010-10-05

**Authors:** Stéphane Tchankouo-Nguetcheu, Huot Khun, Laurence Pincet, Pascal Roux, Muriel Bahut, Michel Huerre, Catherine Guette, Valérie Choumet

**Affiliations:** 1 Unité de Génétique Moléculaire des Bunyavirus, Institut Pasteur, Paris, France; 2 Unité de Recherche et d'Expertise Histotechnologie et Pathologie, Institut Pasteur, Paris, France; 3 Plate-Forme d'Imagerie Dynamique, Institut Pasteur, Paris, France; 4 Université d'Angers, Plate-Forme Technologique de Biotechnologie Moléculaire, Angers, France; 5 Centre de Lutte contre le Cancer Paul Papin, Laboratoire d'Oncopharmacologie, Angers, France; Veterinary Laboratories Agency, United Kingdom

## Abstract

**Background:**

Arthropod borne virus infections cause several emerging and resurgent infectious diseases. Among the diseases caused by arboviruses, dengue and chikungunya are responsible for a high rate of severe human diseases worldwide. The midgut of mosquitoes is the first barrier for pathogen transmission and is a target organ where arboviruses must replicate prior to infecting other organs. A proteomic approach was undertaken to characterize the key virus/vector interactions and host protein modifications that happen in the midgut for viral transmission to eventually take place.

**Methodology and Principal Findings:**

Using a proteomics differential approach with two-Dimensional Differential in-Gel Electrophoresis (2D-DIGE), we defined the protein modulations in the midgut of *Aedes aegypti* that were triggered seven days after an oral infection (7 DPI) with dengue 2 (DENV-2) and chikungunya (CHIKV) viruses. Gel profile comparisons showed that the level of 18 proteins was modulated by DENV-2 only and 12 proteins were modulated by CHIKV only. Twenty proteins were regulated by both viruses in either similar or different ways. Both viruses caused an increase of proteins involved in the generation of reactive oxygen species, energy production, and carbohydrate and lipid metabolism. Midgut infection by DENV-2 and CHIKV triggered an antioxidant response. CHIKV infection produced an increase of proteins involved in detoxification.

**Conclusion/Significance:**

Our study constitutes the first analysis of the protein response of *Aedes aegypti*'s midgut infected with viruses belonging to different families. It shows that the differentially regulated proteins in response to viral infection include structural, redox, regulatory proteins, and enzymes for several metabolic pathways. Some of these proteins like antioxidant are probably involved in cell protection. On the other hand, we propose that the modulation of other proteins like transferrin, hsp60 and alpha glucosidase, may favour virus survival, replication and transmission, suggesting a subversion of the insect cell metabolism by the arboviruses.

## Introduction

Arthropod-borne virus (arbovirus) infections cause a number of emerging and resurgent infectious diseases in humans and animals. Arboviruses are unusual in that they replicate in both arthropod and vertebrate hosts. This leads to a persistent lifelong infection in arthropods and an acute, usually short-duration infection in vertebrates. Traditional means of controlling the spread of arbovirus infection include vaccination of susceptible vertebrates and mosquito control. However, in many cases these measures are either unavailable or ineffective. To successfully implement the strategy of blocking the virus at the insect stage, further knowledge of the virus/vector interactions is required. Studies in this field may identify new genes and possible targets for altering these interactions.

There are three stages that determine the efficiency of an arthropod as an arbovirus vector. The arthropod must ingest a sufficient amount of viremic blood to infect gut cells. After entering gut cells, sufficient replication must then occur for the virus to enter the hemocoel and infect other tissues such as salivary glands, body fat, ovarian tissue, and central nervous chain [1]. In this process, the midgut of mosquitoes is the major barrier to pathogen transmission. This tissue is the environment of viral interaction and replication before dissemination to other organs and tissues. Therefore, key virus/vector interactions and host protein modifications must take place in the midgut for viral transmission to eventually take place [2]. It is now well known that pathogens can remodel and subvert host pathways to facilitate their own survival at the expense of the host [3]. Viruses, as obligate intracellular pathogens with only a limited genome size, are even more dependent on host-encoded factors for their replication cycle [4], [5]. Because some of the host factors are essential for viral growth, they could be useful targets in an anti-pathogen approach.

*Aedes ægypti* is a highly anthropophagic and cosmopolitan species of mosquito. It forms the primary vector of dengue, yellow fever, Chikungunya, and number of other infectious diseases. The *Aedes ægypti* genome of the Liverpool strain has been recently sequenced, and this further facilitates gene identification in this species [6]. Experimental evidence of mosquito gene function in response to pathogens is also becoming available through the use of techniques such as transcriptome analysis by SAGE or microarray, or knockdown of specific gene activity with double-stranded RNA [7], [8], [9], [10]. In contrast to mRNA-based approaches, for which mRNA levels do not always parallel protein levels, proteomics is a definite tool for detecting changes in protein expression and modification. Protein-based approaches have already contributed to the identification of vector proteins reacting to pathogens or endosymbionts [11], [12], [13]. The role of these proteins in the defence of the vector against agression or in the pathogen transmission has been discussed [11], [13]. So far, the only proteomic analyses that have been performed for *Ae. aegypti* have been in larvae brushborder membrane vesicles in response to dengue infection and in non-infected adult female midguts (blood-fed or not) [14], [15]. For *Ae. albopictus*, [16] identified a set of proteins whose expression was increased 24 h after dengue virus infection. These proteins were supposed to be involved in the infection process.

Notwithstanding the central role of the midgut in vector competence, our understanding of how the vector responds to arbovirus infections is very limited. Chikungunya virus (CHIKV) is an alphavirus from the *Togaviridae* family. Dengue 2 virus (DENV-2) is a flavivirus from the *Flaviviridae* family. These two arboviruses are transmitted by *Ae. aegypti*. Alphaviruses and flaviviruses are small enveloped viruses containing plus-sense RNA genomes [17], [18]. The structure, entry and membrane fusion mechanisms have been intensively studied, mainly in vertebrates cells [19], [20], [21]. Our study aims to verify how the same vector respond to different arboviruses at the midgut level and to identify specific or common molecules in the *Ae. aegypti* midgut tissue, which could respond to these two viruses. For this purpose, in the present study we have used 2-Dimensional Differential in-Gel Electrophoresis (2D-DIGE) technology to investigate the proteome of *Ae. aegypti* midguts infected by chikungunya (CHIKV) or dengue-2 (DENV-2) viruses. The putative role of these proteins in pathogen life cycle in the vector will be examined. These results would set a benchmark to which other pathogen/vector interactions may be compared but also would provide clues for the progress in the understanding of the reaction of vectors to pathogens they are able to transmit.

## Results and Discussion

### Follow-up of DENV-2 and CHIKV infections in orally infected *Ae. aegypti* females: IFA and RT-qPCR

**Figure 1:**
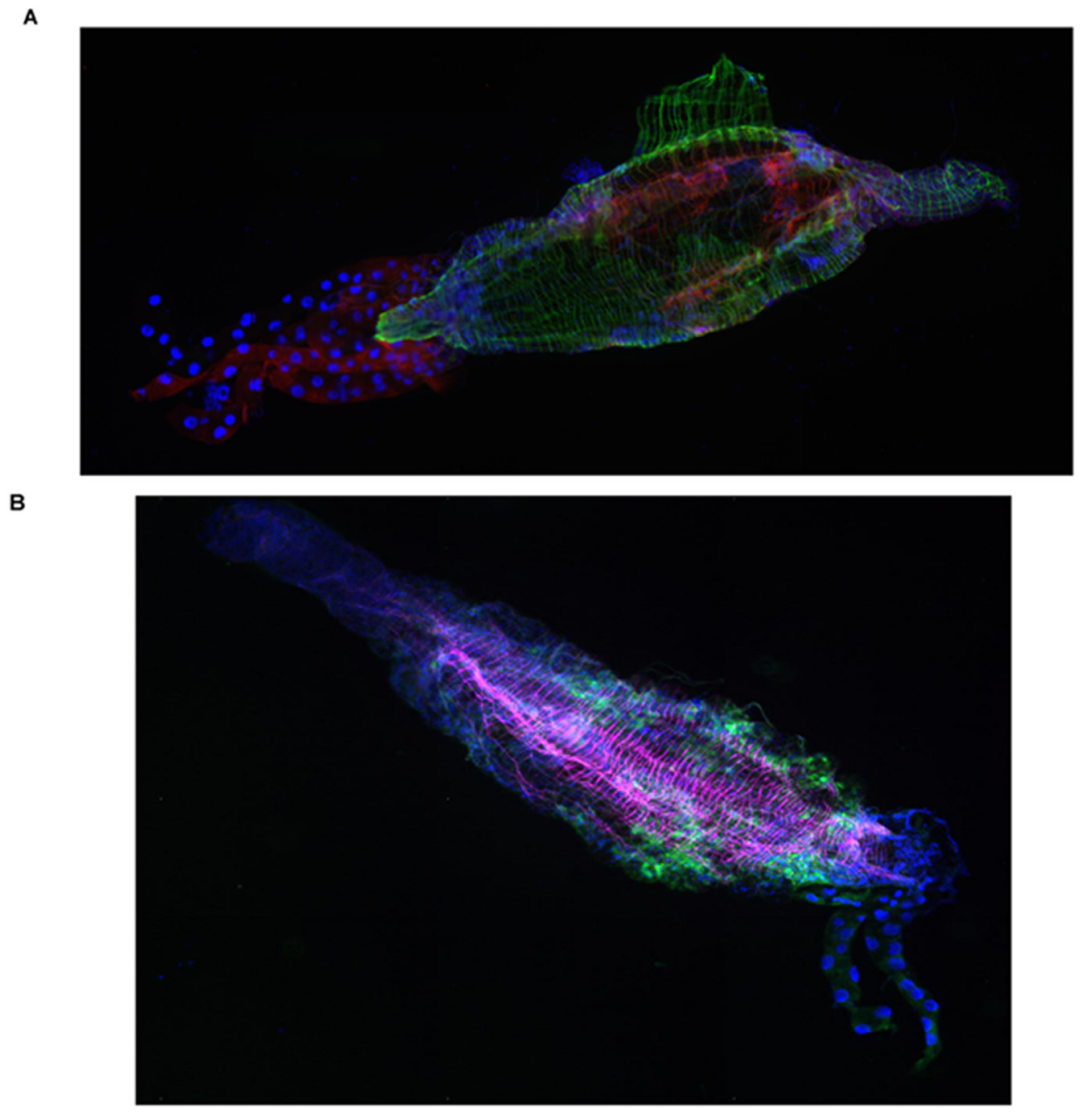
Distribution within the midguts of *Ae. aegypti* after oral infection with CHIKV or DENV-2. *Ae. aegypti* mosquitoes were dissected at 7 DPI and were assayed by IFA to detect CHIKV viral antigen (red) (A) or DENV-2 viral antigen (green) (B). Actin network was labelled using Alexafluor 488 (A) (green) or 633 (B) (magenta) phalloidin. The magnification was 25x.

**Figure 2:**
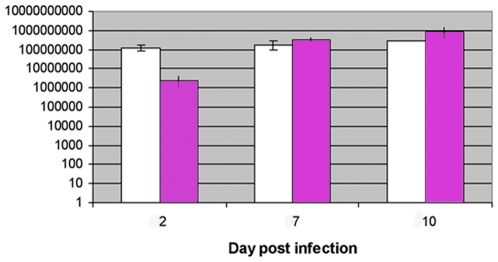
Quantification of CHIKV and DENV-2 RNA by RT-qPCR in infected midguts. The viral RNA copy number was measured for each virus at 2, 7, and 10 DPI.

CHIKV and DENV-2 have different extrinsic incubation periods in *Ae. aegypti* mosquitoes. Depending on the mosquito strain, CHIKV is found in the salivary glands 2 to 4 days after acquisition [22] whereas DENV-2 requires 7 to 14 days to reach this stage [23], [24]. DENV-2 has been reported to reach maximal fluorescence staining in the midgut 7 days after infection of a Chetumal strain [23] whereas no data have been published for CHIKV- infected mosquitoes. To select a time at which the Liverpool strain *Ae. aegypti* midguts were similarly infected by both viruses, we used two different approaches: i) visualization of the distribution of virion particles using IFA, and ii) quantification of viral RNA in the midgut. Figures 1A and B show the distribution of CHIKV and DENV-2 particles in *Ae. aegypti* 7 days post infection (DPI). CHIKV particles are in the anterior part of the midgut whereas DENV-2 particles are in the posterior part. Generally, the intensity of fluorescence appears similar for the two viruses. The imunolocalization of CHIKV and DENV-2 viruses at 7 DPI in mosquito's midgut was determined using histology. Almost all epithelial cells are infected by CHIKV whereas a few patches of them remain uninfected by DENV-2 viruses. In the latter case, however, infected cells are loaded with viral antigens while the anti-CHIKV staining is more pronounced at the apical part of the cells (data not shown). RNA copy number was measured by RT-qPCR for each virus at 2, 7, and 10 DPI (Figure 2). The RNA copy numbers of CHIKV and DENV-2 are similar 7 DPI and remain constant until 10 DPI. We also observed that salivary glands of *Ae. aegypti* (Liverpool strain) were infected at 11 DPI (data not shown).

At 7 DPI, most mosquitoes have developed midgut infection, and this time point is therefore a good marker to investigate modification of mosquito proteins during a persistent infection.

### 2D-DIGE analysis of differential expression in CHIKV and DENV-2-infected midgut

**Figure 3:**
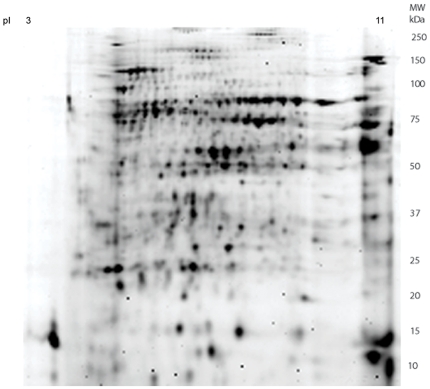
Two-dimensional profile of non-infected *Ae. aegypti* midgut protein extract. The proteins were stained with Cy3. The pI and molecular weight scales are indicated in the Figure.

**Figure 4:**
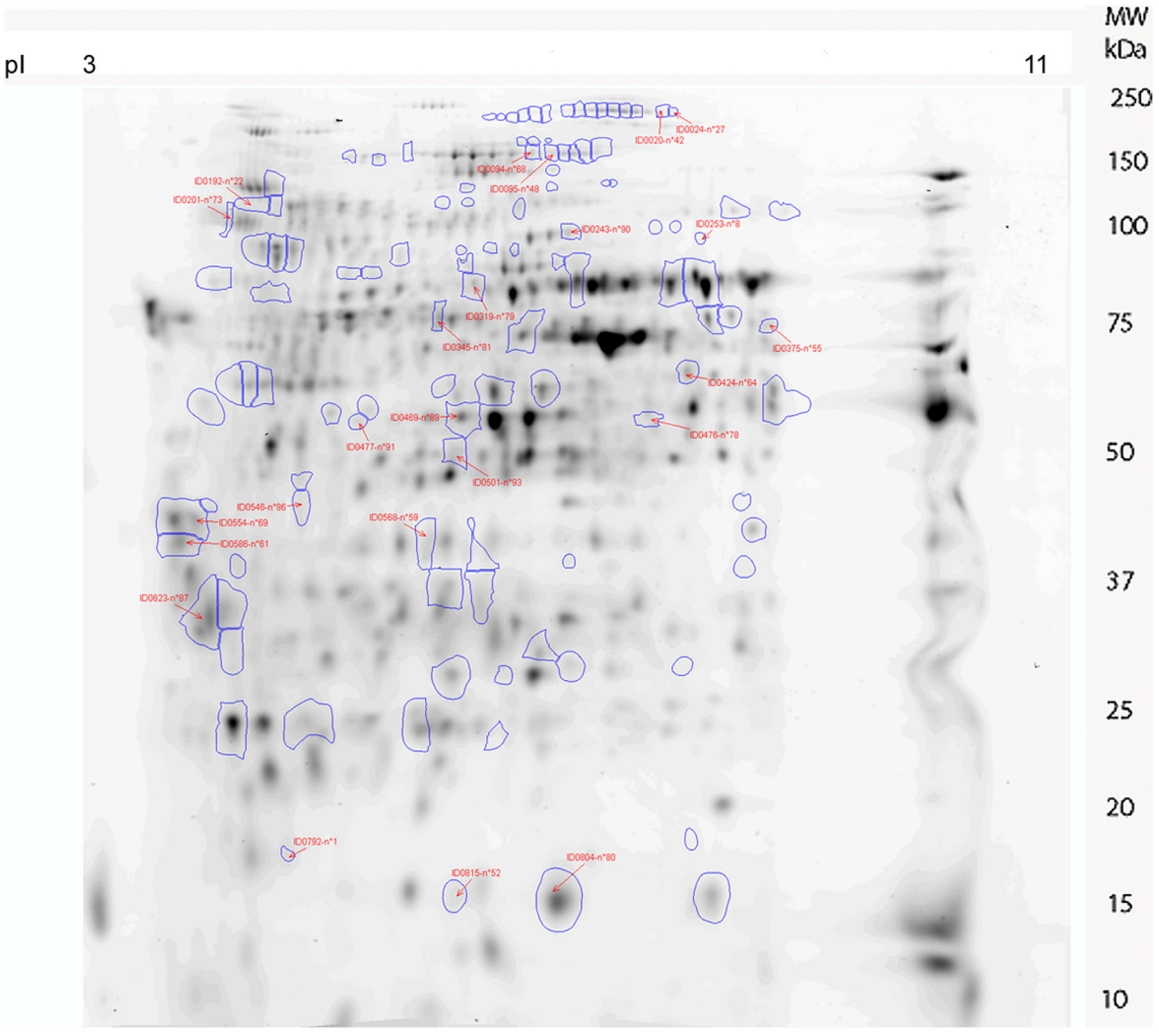
2D-DIGE synthetic gel of *Ae. aegypti* midgut extract modulated in the analysis control/CHIKV infected profiles. Protein spots differentially expressed are indicated by number. The pI and molecular weight scales are indicated in the Figure.

**Figure 5:**
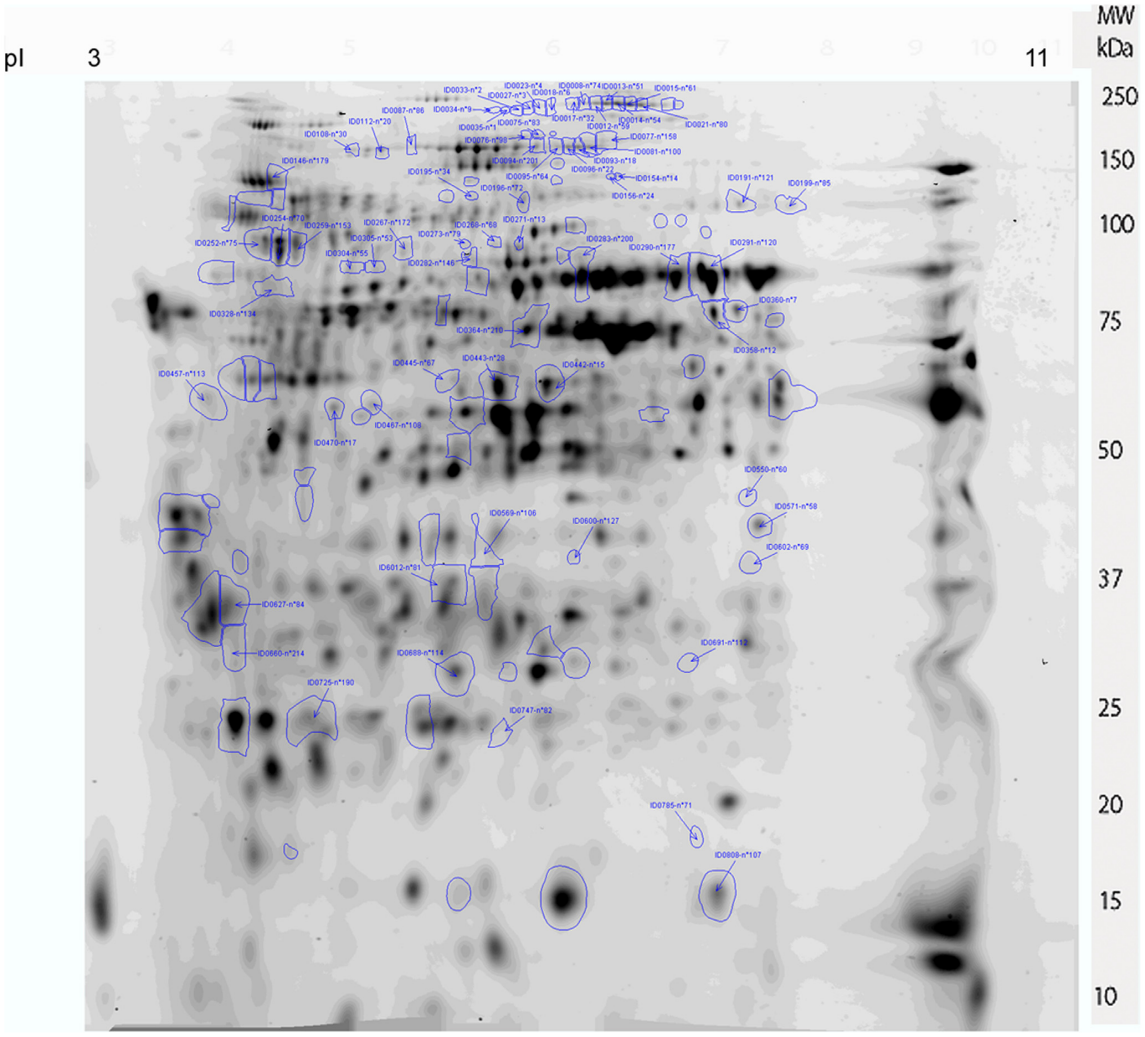
2D-DIGE synthetic gel of *Ae. aegypti* midgut extracts showing spots modulated after analysis of control/DENV-2 profiles. Protein spots differentially expressed are indicated by number. The pI and molecular weight scales are indicated in the Figure.

2D-DIGE electrophoresis (2-Dimensional Differential in-Gel Electrophoresis) is the most reliable and reproducible technique of comparative proteomics [25]. It is based on the special properties of fluorescent probes: the CyDyes. Two protein extracts with distinct probes can be loaded on the same gel. In addition, an internal standard labelled with the third probe is incorporated to the gel, allowing a normalization of abundance ratios to provide multivariable experiments with great statistical power. Virtual elimination of gel variation allows the identification of induced biological changes with statistical accuracy capable of revealing differences in abundance of less than 10% between samples, thereby allowing a low threshold of significant fold modulation that we set at 1.3. Three independent infections were performed with each virus in parallel with six controls where artificial feeding was performed with non-infected blood. Pools of 100 to 150 midguts were collected after each experiment and the same amount of protein extracts from each pool (50 µg) were used for the DIGE experiments. Six gels were run according to the experimental protocol described in Table S1. Images of the six gels showing control and CHIKV/DENV-2 infected midgut extract profiles are shown in Figure S1. Analysis of the gel with Progenesis SameSpots software (Nonlinar dynamics) allowed the detection of 860 spots per gel. A control gel is shown in Figure 3. Four analyses were performed from these gels: i) comparing control profiles with CHIKV and DENV-2 infected profiles (control/CHIKV/DENV-2); ii) comparing control with CHIKV infected samples (control/CHIKV); iii) comparing control with dengue infected profiles (control/DENV-2), and iv) comparing DENV-2 and CHIKV infected profiles (DENV-2/CHIKV). A total of 113 variant spots were excised from the gels, digested by trypsin and analyzed by MALDI-TOF/TOF mass spectrometry (Figure S2; Table S2). Thirty-two spots were not identified in the database. For each of the four comparative analyses, the following numbers of spots were identified: control/CHIKV, 24 spots (Figure 4), control/DENV-2, 68 spots (Figure 5); control/CHIKV/DENV-2 comparison, 54 spots (Figure S3); DENV-2/CHIKV, 42 spots (Figure S4).

A list of the proteins identified in these spots by mass spectrometry is shown in Table S2. Table S3 indicates the identification number, the modulation of protein expression observed in the various comparisons, their spot number in the corresponding gel and the putative localization and function of the proteins. Table S4 shows the modulation triggered by each virus for each identified protein from all of the gel data comparisons listed according to their putative function. The control/DENV-2/CHIKV, control/DENV-2 and control/CHIKV comparisons show that 18 proteins are modulated by DENV-2 exclusively and 12 proteins are modulated by CHIKV exclusively (Table S4). Both viruses influenced the expression of 11 proteins in the same manner (up-regulation or down-regulation). However, the level of 9 proteins was influenced differently by each virus. In the DENV-2/CHIKV comparison, another 9 proteins were differentially regulated.

We then investigated the modulation of protein expression related to putative function after CHIKV and DENV-2 infection.

#### Proteins involved in oxidative stress

Several studies have reported that viral infections cause oxidative stress through the release of reactive oxygen species (ROS) [26], [27]. In our study, 2-D gel electrophoresis investigations have shown several spots of aldehyde oxidase, a ROS-generating enzyme, suggesting post-translational modeling of this protein, possibly by phosphorylation. Indeed, the high polymorphism of aldehyde oxidase in *Drosophila* has been proposed to be due to posttranslational modifications [28]. In the present study, expression of aldehyde oxidase was increased in CHIKV and DENV-2 infected midguts (Table S4). Increased oxidative stress in the host may cause direct damage to the viral RNA itself, causing new mutations that may lead to enhanced pathogenesis. However, under normal conditions, ROS produced by metabolism are removed by a series of antioxidant enzymes.

#### Antioxidant related proteins

Antioxidants are molecules that neutralize free radicals by accepting or donating an electron to remove unpaired electrons. The first line of natural antioxidant defence is provided by three types of primary antioxidant enzymes that act directly on ROS: superoxide dismutases, catalase, and peroxidases. In addition, insects have three families of genes that encode peroxidase antioxidant enzymes [29]: TPXs, also known as peroxiredoxins [30], phospholipid-hydroperoxide GPX homologs with thioredoxin peroxidase activity (GTPX) [31], and glutathione S-transferases (GSTs) [32], [33]. Secondary antioxidant enzymes act indirectly on ROS and include TrxR, which recycles both TRX and GSH [34].

DENV-2-infected midguts showed increases of TrxR, catalase and malic enzyme, whereas, CHIKV-infected midguts showed an increase of peroxiredoxins and GST (Table S4). This indicates that the responses in the mosquito midgut to each virus were dissimilar. Malic enzyme catalyzes the interconversion of L-malate and oxaloacetate with nicotinamide adenine dinucleotide (NAD) as a coenzyme. This reaction produces reduced nicotinamide adenine dinucleotide phosphate (NADPH), which is crucial to cellular anti-oxidative defence strategies in most organisms. Ten days after DENV-2 infection, Xi *et al.* [35] observed down-regulation of several oxidative defence enzymes in midguts on microarray analysis. These included TPX2, TPX3, TPX4, and catalase. In contrast, we report an up-regulation of catalase expression, but this may be explained by the earlier collection of midguts in our experiments. GST has been implied in the defense of *Anopheles* mosquito to parasite infection [36]. According to this study, silencing homologs of glutathione-S-transferase theta (GSTT) in *A. gambiae* has been shown to significantly reduce *Pl. berghei* infection. Although anti-parasite and anti-viral defense systems in the mosquito may differ due to their cell interactions and lifestyles, it could be of interest to investigate the effects of repressing GST genes in CHIKV infection.

#### Proteins involved in cell detoxification

Infection of midguts was also shown to modulate three enzymes involved in cell detoxification. Of these, lactoyl glutathione lyase was down-regulated in CHIKV and DENV-2 infected midguts, whereas alcohol dehydrogenase and aldo keto reductase were up-regulated only in CHIKV-infected midguts. Alcohol dehydrogenase facilitates the conversion of toxic alcohols to aldehydes and aldo keto reductase is involved in the protection of cells from endogenously formed reactive carbonyl groups. Both of these actions are in favour of cell survival.

#### Proteins involved in energy production

In eukaryotic cells, several metabolic pathways are involved in energy production. These include the glycolytic pathway, the tricarboxylic acid cycle, and the pentose phosphate pathway. We found that various enzymes participating in these energetic pathways were modulated by CHIKV and DENV-2 infections. Several enzymes involved in the glycolytic pathway were up-regulated by CHIKV or DENV-2 (Table S4), suggesting extensive glucose utilization during midgut infection. Recent studies have demonstrated that some of these enzymes are multifaceted proteins rather than simple components of the glycolytic pathway. One of them, enolase, is involved in transcriptional regulation [37] and was shown to stimulate transcription of the Sendai virus genome [38], but it is unclear whether this is due to its glycolytic activity or an alternative function.

#### Proteins involved in carbohydrate metabolism

Alpha-glucosidase and beta-galactosidase are both involved in carbohydrate metabolism. While alpha-glucosidase was up-regulated in DENV-2 infection, beta-galactosidase was up-regulated in CHIKV infection (Table S4). Alpha-glucosidase inhibitors have been shown to eliminate the replication of several endoplasmic reticulum-budding viruses like DENV-2 [39], [40], emphasizing on an important role of this enzyme in DENV-2 infection in mosquito midgut cells.

#### Proteins involved in lipid metabolism

Concerning enzymes involved in lipid metabolism, Acyl CoA transferase was up-regulated by both types of viruses, whereas 4 hydroxybutyrate CoA transferase was up-regulated by DENV-2 and 3-hydroxyacyl-coA dehydrogenase was up-regulated by CHIKV (Table S4). 4 hydroxybutyrate CoA transferase is involved in the butyrate biosynthetic pathway and butyric acid fermentation is the preferred energy source in the gut wall of vertebrates [41]. Fatty acids are also an important source of energy for the cell and Acyl CoA transferase 4 hydroxybutyrate and 3-hydroxyacyl-coA dehydrogenase are both involved in mitochondrial fatty acid oxidation. Samsa *et al*. [42] have reported that dengue virus infection increases the number of lipid droplets per cell, suggesting a link between lipid droplet metabolism and viral replication. Indeed, interfering with lipid droplet formation/metabolism with a fatty acid synthase inhibitor (C75) inhibited viral particle formation by over 1000-fold. Thus, modulation of fatty acid catabolism could be detrimental to virus formation and could be a cellular mechanism to both produce energy and fight against a high replication of DENV-2.

#### Proteins involved in amino acid and protein metabolisms

Many proteins involved in protein and amino acid metabolism and modification were modulated by both viruses (Table S4). Metalloproteases and aminopeptidases were down regulated by DENV-2. Glutamyl aminopeptidase, an enzyme up regulated by both viruses, is thought to be involved in a major degradation pathway of proctolin, an insect neuropeptide which acts as a potent stimulator in the contraction of visceral and skeletal muscles. In *Ae. aegypti*, glutamyl aminopeptidase has been reported to be down-regulated in response to the stress of heat shock treatment [43]. In the present study, the observed down-regulation of glutamyl aminopeptidase may modify the vector's behavior by interfering with the degradation of proctocolin.

We showed that protein disulfide isomerase (PDI) was up-regulated in CHIKV infected midguts and down-regulated in DENV-2 (Table S4). PDI is a multifunctional protein that catalyzes thiol–disulfide interchanges underlying the formation, reduction, and rearrangement of secreted and cell-surface-associated proteins [44]. PDI has been demonstrated to play a role in redox control at the cell surface [45]. In response to increased extracellular reduction, PDI may help to re-establish redox homeostasis by rearranging and forming disulfide bonds, thereby protecting the cell against this aggression [46]. PDI is also an essential component of the endoplasmic reticulum, which is involved in viral translation, replication, and encapsidation. In particular, PDI has been located by [47] in the complex I, the main ribonucleoprotein complex formed with the 3′UTR in dengue 4 virus replication. It is therefore likely that PDI plays a role in viral replication, translation, or encapsidation, and modulation of the expression of this protein would interfere with viral replication.

The level of Hsp60 was up-regulated in *Ae. aegypti* midguts infected with DENV-2. In agreement with this observation, [48] have recently reported that RNA interference mediated silencing of Hsp60 gene in human monocytic myeloma cell line U937 revealed decreased dengue virus multiplication. This suggests that Hsp60 protein interferes positively with dengue virus infection. In contrast, the transcription of Hsp60 was shown to be down-regulated in midguts infected with an alphavirus, Sindbis virus, 8 DPI, whereas our study did not show any modulation of the protein level by CHIKV [8]. Once again, these results show a diferential modulation of protein level by viruses belonging to different families.

#### Proteins involved in translation machinery

The level of elongation factor 1 (EF1) gamma is higher in CHIKV infected midguts than control midguts. It is the most highly modulated protein of all CHIKV modulated proteins identified in our study. In eukaryotes, the soluble elongation factor EF1 is composed of three or four subunits, EF1 alpha,-beta, -gamma, and -delta in higher eukaryotes and is required for translational elongation. EF1 gamma has been implicated in the innate immune response of Drosophila and was found essential to cell viability [49], [50]. We can therefore postulate that in *Ae. aegypti* midgut, increase of EF1 gamma expression may play a role for protecting cell against CHIKV.

#### Proteins involved in iron transport and storage

The protein transferrin is involved in iron transport and was down-regulated both in CHIKV and DENV-2 infections. Iron transport and storage proteins have diverse roles in insect physiology. The host's ability to sequester iron and hinder pathogen survival is of interest for innate immunity. There have been several reports, describing up-regulation of transferrins in insects or insect cells challenged with bacteria [51], suggesting an antibacterial role of this protein. The *D. melanogaster* transferrin gene contains promotor region sequences known to bind nuclear factor-kappa B–like transcription factors which are involved in the insect immune response [52]. Therefore, a down-regulation of transferrin may favour viral multiplication.

#### Proteins involved in cell cytoskeleton

Viral infection altered the expression of some proteins involved in cell cytoskeleton and cytoplasmic transport (Table S4). Actin expression was higher in DENV-2 infection than in CHIKV infection whereas the reverse was observed for moesin, a cytoskeletal binding protein involved in microtubule organization. Cofilin, an actin depolymerising factor, was down-regulated in DENV-2 infection, but up-regulated in CHIKV infection. Calponin, a shape change sensitive actin binding protein was down-regulated in DENV-2 infection. It was also down-regulated in cells infected by cytomegalovirus [53]. These observations suggest that different cytoskeletal modulations are occurring after midgut infection by either virus and may explain in part the pathological changes in mosquito midgut epithelial cells observed after arbovirus infection [54].

#### Host protein that may be incorporated into viral particles

In a previous study performed with influenza virus, several host proteins were shown to be incorporated into the viral particle [55], an observation also reported for other enveloped viruses such as poxviruses, retroviruses, and herperviruses. These include both cytoplasmic and membrane-bound proteins that can be grouped into several functional categories, such as cytoskeletal proteins, annexins, glycolytic enzymes, and tetraspanins. Therefore, as well as being due to host responses, protein levels in our study may have changed because of their incorporation into virus particles, since as reported above, we observed that some of the previously identified host proteins incorporated into viral particles (enolase, aldoketoreductase, peroxiredoxin 1, annexin, actin, cofilin, wild type (WD) repeat containing protein, and transgelin) were specifically modulated by either CHIKV or DENV-2 infection (Table S4). These proteins could have also been included into the viral particles of DENV-2 or CHIKV. Both viruses are enveloped viruses and must enter the cell via a membrane fusion event and leave the cell by budding, either from the plasma membrane or an internal membrane. It is possible that the incorporated host proteins that are common to enveloped viruses play a role in these particular stages of the virus life-cycle. In addition, the host cytoskeletal network is involved in the transport of viral components in the cell, especially during the stages of virus entry and exit from the cell [56], [57]. Several studies on RNA viruses have indicated that cytoskeletal proteins such as actin are also required for viral gene expression. The putative presence of these proteins in viral particles could reflect their active participation in moving viral components to the assembly site as well as cytoskeletal reorganization that occurs during bud formation. Other proteins associated with a particular virus included annexins and WD repeat containing protein, both of which were up-regulated in CHIKV infection. Annexins are calcium dependent phospholipid-binding proteins and have been suggested to act as scaffolding proteins at certain membrane domains. For example, annexin A2 is required for the apical transport of vesicles in polarized cells, specifically vesicles that carry membrane raft-associated proteins [58].

In conclusion, our study shows that CHIK and DENV-2 are both able to modulate the expression of several mosquito's midgut proteins belonging to a variety of functional groups. These include structural (cytoskeleton), redox, regulatory proteins, and enzymes for several metabolic pathways. Both viruses induce an overexpression of proteins involved in cell protection. This is especially the case for proteins involved in the antioxidant response and in detoxification. These proteins play an important functional role in *Ae. aegypti*, perhaps enhancing survival during infection. On the other hand, they also modulate the expression of other proteins, like transferrin (CHIKV and DENV-2), hsp60 and alpha glucosidase (DENV-2), which may favour virus survival and replication inside the midgut. These results suggest a subversion of the insect midgut metabolism by the arboviruses. DENV-2 induced the modulation of more midgut proteins than CHIKV. This observation might be explained by different virus/host cell interactions during virus life cycles. However, the different speed of dissemination of the two arboviruses from the midgut might also explain the differential modulation observed in this organ after infection. At 7 DPI, DENV-2 has just started to escape midgut whereas CHIKV has already reached the salivary glands. The timing of vector response to the arboviruses may vary as function of their extrinsic period, a steady state being reached earlier for those which disseminate more rapidly. These observations give further emphasis to the interest of studying the interactions between the arthropod host and the pathogen it transmits. Investigations using RNAi in the Liverpool strain as well as in field-collected *Ae. aegypti* mosquito, should be the next step to assess the role of these proteins in viral replication and dissemination within the mosquito *Ae. aegypti*.

## Materials and Methods

### Mosquitoes

*Aedes aegypti* (Liverpool strain) eggs were kindly provided by Prof. D. Severson (department of Biological Science, Notre Dame University, Wisconsin-Madison, USA). They were maintained at 28±1°C under 80% relative humidity with a light/dark cycle of 16 h/8 h. Larvae were reared in pans containing cat food (beef and chicken) in 1 L of tap water. Adults were provided with 10% sucrose solution *ad libitum*.

### Viruses

The CHIKV 06.21 isolated in November 2005 from a new-born male from La Reunion presenting meningo-encephalitis symptoms was used for all experiments [59]. This strain contained the change A→V at the position 226 in the E1 glycoprotein (E1-226V). Stock virus was produced following passages on *Ae. albopictus* C6/36 cells then harvested and stored at −80°C in aliquots as described by [60]. The titre of the frozen stock virus was estimated to 10^9^ plaque-forming units (PFU)/mL.

The dengue 2 virus strain, provided by Leon Rosen (Institut Pasteur, Paris, France) was isolated in 1974 from a human serum in Bangkok (Thailand) (D2BN32). Viral stocks were produced by inoculating *Ae. albopictus* cells (C6/36 clone) with triturated infected mosquitoes. The mosquito cells were maintained as described in [61]. Titration of the virus stock was carried out in *Ae. aegypti* (Paea strain) by inoculating serial dilutions of the supernatant intra-thoracically. Mosquito infection was detected by an IFA assay on head squashes. Titres were calculated by the 50% endpoint method [62] and expressed as 50% mosquito infectious doses (MID_50_/ml). The titre of the stock virus was estimated to 10^10.2^ MID_50_/ml.

### Oral infections of mosquitoes and dissections

Seven day-old female mosquitoes were deprived of sucrose 24 h prior the infectious blood meal. They were then allowed to feed for 15 min through a chicken skin membrane covering glass feeders maintained at 37°C. The infectious blood meal was comprised of two thirds washed rabbit erythrocytes, one third viral suspension, and ATP (as a phagostimulant) at a final concentration of 5×10^-3^ M. The infectious blood was at a titre of 10^7.5^ PFU/ml (CHIKV 06.21) or 10^9^ MID_50_/ml (DENV-2).

### Preparation of midgut protein extracts

Pools of 30 entire midguts were dissected in 30 µl phosphate buffered saline (PBS) containing protease inhibitors (Complete, Roche Diagnostics) 7 days after feeding. These were kept at -80°C until use. Midguts were disrupted by ultrasound (Cup Horn, Sonics & Material) for 30 min with 2 sec pulse on and 2 sec pulse down, at maximum amplitude. Midgut homogenates were then centrifuged for 30 min at 130,000 g and proteins were quantified using the BCA protein assay (Pierce). Aliquots of midgut proteins were then lyophilized and resuspended in 2D-DIGE buffer.

### Reverse transcription quantitative PCR (RT-q PCR)

Total RNA from mosquitoes or midguts was extracted using the Nucleospin® RNA II kit (Macherey-Nagel) according to the manufacturer's instructions. RNA was eluted in 40 µl of RNAse-free H_2_O by centrifugation at 11,000 g for 1 min.

Synthetic RNA transcripts for CHIKV and DENV-2 were generated to construct a standard curve. The targeted region in the CHIKV sequence was amplified by PCR product and ligated into pCR II TOPO vector (Invitrogen). The plasmid was then linearized using EcoRI restriction enzyme and purified using QIAquick PCR purification kit. RNA transcripts were prepared *in vitro* using the RiboMAX™ Large Scale RNA Production Systems (Promega) appropriate for either SP6 or T7 RNA polymerase. The transcript size was 1,356 bp. Residual DNA was been eliminated with several DNAse treatments (Turbo DNA-free (Ambion)). After quantification by spectrophotometer, RNA transcript solution was stored at −80°C.

The one-step reverse transcription quantitative PCR (RT-qPCR) was performed using Power Sybr Green RNA-to-Ct one step kit (Applied Biosystems). CHIKV primers were selected in the E2 structural protein regions: sense Chik/E2/9018/+ (CACCGCCGCAACTACCG); anti-sense Chik/E2/9235/- (GATTGGTGACCGCGGCA). DENV-2 primers were selected as described in Lanciotti *et al*., 1992: sense CGCCACAAGGGCCATGAACAG, antisense (TCAATATGCTGAAACGCGCGAGAAACCG).

RT-qPCR was performed using Applied Biosystem's Fast Real-Time PCR Systems 7500 with the software 7500 v.2.0.1. The thermal cycling conditions included: a reverse transcription step at 48°C for 30 min, an inactivation step of RT/RNAse enzyme at 95°C for 10 min followed by 40 cycles of 95°C 15 s, 60°C 1 min, a final denaturation step where the temperature increases from 60°C to 95°C during 20 min and a step of 15 sec at 95°C. Signals were normalized to the standard curve using serial dilutions of RNA synthetic transcripts. Using ΔC_t_ analysis, normalized data were used to estimate the transcript copy number in infected mosquitoes.

### Immunofluorescence (IFA)

After dissection, midguts were placed on a slide. PBS was removed and midguts were fixed in 4% paraformaldehyde (PFA) for 1 h, dried and kept at 4°C until use. For indirect fluorescent-antibody (IFA) experiments, midguts were rehydrated in PBS for 3×5 min, and then incubated for 1 h with Triton X100 (0.2%). They were then washed again with PBS (3×5 min) and incubated for 30 min with PBS containing 5% BSA. The slides were drained and incubated overnight at 4°C with anti-DENV-2 protein E 3H5 diluted 1∶400 in PBS, then washed with PBS (3×5 min) under shaking. They were incubated for 1 h with 1∶500 Alexafluor 488 goat anti/mouse (Invitrogen), and washed with PBS. Actin network was stained with Phalloidin Alexafluor 633 or 488 (Invitrogen) (diluted 1/40 in PBS). After washing, a drop of Prolong gold antifade (Invitrogen) was settled on each slide and a coverslide was added. All preparations were examined by confocal microscopy (Zeiss LSM 510 Meta and TCS SP5 Leica Microsystems).

### Histological observations

At 7 DPI, 5 infected and control females were killed and fixed in Carnoy solution (3 vol. chloroform, 1 vol. absolute ethanol, 1 vol. acetic acid). Samples were then dehydrated as follows: 8 h in absolute ethanol, 17 h in solution 1 (55% n-butanol/40,5% absolute ethanol in H2O), 8 h in solution 2 (75% n-butanol/22.5% absolute ethanol in H2O) and finally 2–3 days in n-butanol. Mosquitoes were embedded in paraffin. Sections (5 µm) were stained with hematoxylin and eosin, periodic acid Schiff, and Gordon sweet stains according to [63]. Immunohistochemical analysis was performed by using a anti-CHIKV or DENV-2 polyclonal mouse ascitic fluid at a dilution 1∶750. Briefly, tissue sections were immersed in 200 mL of citrate and incubated three times for 5 min in a microwave at 650 W before staining. The streptavidin peroxydase method with AEC (amino ethyl carbozole) as a chromogen was used to detect the secondary antibody (Envision system labeled Polymer-HRP antimouse, Dako). Slides were counterstained with Meyer's hematoxylin. Slides were observed with light microscopy.

### 2D-DIGE

Midgut protein extracts from infected and non-infected mosquitoes were compared in individual 2D gels to identify proteins unique to or significantly modulated in presence of each virus. For this experiment, three distinct infection experiments were performed with each virus. For each experiment, control mosquitoes were fed on non-infected red cells. Extracts were prepared from infected and non-infected midguts, dissected 7 DPI.

Fluorescent CyDye-labelling of proteins for DIGE was performed according to the manufacturer's instructions (GE Healthcare Bio-Sciences Corp.). Proteins from a pair of protein extracts (50 µg total proteins/gel) were labeled with Cy3 or Cy5 (4 pmol/µg proteins) and corun on the same gels. A dye swap was used to normalize for any bias introduced by the dyes, since different fluorescent CyDyes may have different efficiencies in labelling different proteins. An internal standard was prepared containing a mixture of equal amounts of proteins from each extract. This standard was labeled with Cy2 (8 pmol/µg proteins) and was included in each gel for between gel comparisons on image analysis software. The protein samples were loaded onto immobilized pH gradient (IPG) strips (pH 3 to 11 nonlinear, 18 cm). The first dimension was run on the IPGphore III (GE Healthcare Bio-Sciences Corp.) at 20°C with the following settings: step 1, 500 V, 1 h; step 2, 500 V to, 1,000 V, 4 h; step 3, 1000 V to 8,000 V, 3 h, step 4: 8000 V, 1 h. Before the second dimension was run, the strips were reduced for 10 min with 64.8 mM of dithiothreitol in sodium dodecyl sulfate (SDS) equilibration buffer (50 mM Tris-HCl [pH 8.8], 6 M urea, 30% glycerol, 2% SDS, 0.002% bromophenol blue) and then alkylated for 15 min with 135.2 mM of iodoacetamide in the same equilibration buffer. The second dimension was carried out in the Ettan DALT Six system (GE Healthcare Bio-Sciences Corp.) at 25°C in an electrode buffer (25 mM Tris, 192 mM glycine, and 0.1% [wt/vol] SDS) with the following settings: step 1, 2 W/gel, 25 min; step 2, 17 W/gel, 4 h. The gels used in the second dimension were 12.5% homogenous acrylamide gels cast in the laboratory. Immediately after electrophoresis, the gels were scanned with a Ettan DIGE Imager (GE Healthcare Bio-Sciences Corp.). Three replicates of each infected sample and 6 replicates for control extracts (non-infected) were used. The gel images were initially analyzed by Progenesis SameSpots, V2.0 (Nonlinear USA Inc., Durham, NC). To identify the proteins associated with DENV-2 and CHIKV infections, the SameSpots image analysis software was used. To analyze the numbers of spots regulated by the viral infection or specific spots were determined by spot-by-spot and gel-by-gel manual confirmation on all the 2D and 3D images for the group. The spots reported all had at least 1.3-fold intensity (normalized to volume) difference, and all were statistically significant, which was measured with the built-in statistical tool in the SameSpots software (Anova, p<0.05).

### Protein identification

Spots that were unique to or clearly up-regulated or down-regulated in infected or non-infected mosquitoes were recovered using the Ettan Spot picker (GE Healthcare) and individually treated with the Proteoextract All-in-one Trypsin Digestion kit (Calbiochem) according to manufacturer's instructions. Peptide digests were concentrated on C18-Zip Tips (Millipore) and eluted with 1.5 µl α-cyano-4-hydroxycinnamic acid matrix solution (in 50% acetonitrile, 0.05% trifluoroacetic acid) onto a MALDI-TOF target plate (Opti-TOF 384 well Insert, Applied Biosystems). Peptide spectra acquisition was realized on the 4800 MALDI TOF/TOF Analyzer (Applied Biosystems). After screening the sample position in MS-positive reflector mode using 1500 laser shots, the fragmentation of automatically-selected precursors was performed at collision energy of 1 kV using air as collision gas (pressure of 2×10^-6^ Torr). MS spectra were acquired between m/z 800 and 4000. Up to 12 of the most intense ion signals having a signal to noise ratio >12 were selected as precursors for MS/MS acquisition. Peaklist generation and protein identification were performed by the ProteinPilotTM Software V 2.0 (Applied Biosystems) using the Paragon algorithm. Each MS/MS spectrum was searched for all species against the Vectorbase database. The searches were run with the fixed modification of iodoacetamide labeled cysteine parameter enabled. Other parameters such as tryptic cleavage specificity, precursor ion mass accuracy and fragment ion mass accuracy are MALDI 4800 built-in functions of ProteinPilot software. The ProteinPilot software calculates a confidence percentage (the unused score), which reflects the probability that the hit is a false positive, meaning that at 95% confidence level, there is a false positive identification chance of about 5%.

The preliminary protein identifications obtained automatically from the software were inspected manually for conformation prior to acceptance.

## Supporting Information

Table S1 Experimental procedure for 2D-DIGE.
(0.03 MB DOC)

Table S2 List of proteins identified by mass spectrometry.
(0.88 MB DOC)

Table S3 Summary of proteins identified as being modulated after midgut infection by CHIKV or DENV-2.
(0.32 MB DOC)

Table S4 Differential expression of midgut proteins according to their role after infection by CHIKV or DENV-2 viruses.
(0.15 MB DOC)

Figure S1 2D-DIGE gels run with control, CHIKV- and DENV-2- infected *Ae. aegypti* midgut extracts. Pools of 50 µg of control blood-fed, CHIKV blood-fed and DENV-2 blood-fed midgut extracts were processed by 2D-DIGE. The gels were performed according to the protocole described in Table S1.
(7.66 MB TIF)

Figure S2 2D-DIGE synthetic gel of *Ae. Aegypti* midgut extracts. Protein spots differentially expressed by both viruses are indicated by number.
(6.04 MB TIF)

Figure S3 2D-DIGE synthetic gel of *Ae. aegypti* midgut extracts showing spots modulated after analysis of control/CHIKV/DENV-2 profiles. Identification numbers (ID) and the range of each spot is shown on the gel. The pI and molecular weight scales are indicated in the Figure.
(5.45 MB TIF)

Figure S4 2D-DIGE synthetic gel of *Ae. aegypti* midgut extracts showing spots modulated after analysis of CHIKV/DENV-2 profiles. Identification numbers (ID) and the range of each spot is shown on the gel.
(5.24 MB TIF)
