# Supplementary material for: Differential Protein Modulation in Midguts of Aedes aegypti Infected with Chikungunya and Dengue 2 Viruses
Source: PLoS One. 2010 Oct 5;5(10):e13149. doi: 10.1371/journal.pone.0013149 (PMC2950154; doi:10.1371/journal.pone.0013149)
Supplement: Table S1 — Experimental procedure for 2D-DIGE. (0.03 MB DOC) [file pone.0013149.s001.doc]

Table S1: Experimental procedure for 2D-DIGE

|  | Cy2 | Cy3 | Cy5 |
| --- | --- | --- | --- |
| Gel 1 | IS | Control extract 1 | DENV-2 extract 1 |
| Gel 2 | IS | Control extract 2 | Control extract 3 |
| Gel 3 | IS | CHIKV extract 1 | Control extract 4 |
| Gel 4 | IS | CHIKV extract 2 | DENV-2 extract 2 |
| Gel 5 | IS | DENV-2 extract 3 | Control extract 5 |
| Gel 6 | IS | Control extract 6 | CHIKV extract 3 |

Three infection experiments were performed with each virus, with control non-infected blood-fed mosquitoes for each experiment. Protein extracts were prepared from pooled midguts corresponding to each experiment. IS: internal standard.
