# Supplementary material for: Differential Protein Modulation in Midguts of Aedes aegypti Infected with Chikungunya and Dengue 2 Viruses
Source: PLoS One. 2010 Oct 5;5(10):e13149. doi: 10.1371/journal.pone.0013149 (PMC2950154; doi:10.1371/journal.pone.0013149)
Supplement: Table S2 — List of proteins identified by mass spectrometry. (0.88 MB DOC) [file pone.0013149.s002.doc]

Table S2 : List of proteins identified by mass spectrometry

| **N°ID** | **Unused** | **Accession (Vectorbase)** | **Accession (SwissProt)** | **theo prot MW (kDa)** | **theo prot pI** | **Name** | **Peptide number** | **Conf (%)** | **Peptide sequences** | **Modifications** |
| --- | --- | --- | --- | --- | --- | --- | --- | --- | --- | --- |
| **0008** | 3.53 | AAEL010382-PA | Q16T47_AEDAE | 139.591 | 6,28 | aldehyde oxidase | 4 | 97.00 | VPQNSLNLYVR |  |
|  |  |  |  |  |  |  |  | 88.00 | NLFENAVVQEALK |  |
|  |  |  |  |  |  |  |  | 70.00 | LQVFVDVSSIEELR |  |
|  |  |  |  |  |  |  |  | 46.00 | SLAYDAEPR |  |
| **0012** | 9.7 | AAEL010382-PA | Q16T47_AEDAE | 139.591 | 6,28 | aldehyde oxidase | 5 | 99.00 | ACEIILER | Carbamidomethyl(C)@2 |
|  |  |  |  |  |  |  |  | 99,00 | FLQNSTNATGVLR |  |
|  |  |  |  |  |  |  |  | 99,00 | HIDLIANVPVR |  |
|  |  |  |  |  |  |  |  | 99.00 | SLAYDAEPR |  |
|  |  |  |  |  |  |  |  | 98.00 | LAGNSVEHFTLN |  |
| **0013** | 6.44 | AAEL010382-PA | Q16T47_AEDAE | 139,591 | 6,28 | aldehyde oxidase | 4 | 99.00 | AQNAHAYVNAAFLLK |  |
|  |  |  |  |  |  |  |  | 99.00 | HIDLIANVPVR |  |
|  |  |  |  |  |  |  |  | 96,00 | FLQNSTNATGVLR |  |
|  |  |  |  |  |  |  |  | 91.00 | NLFENAVVQEALK |  |
| **0014** | 14.3 | AAEL010382-PA | Q16T47_AEDAE | 139,591 | 6,28 | aldehyde oxidase | 8 | 99.00 | ACEIILER | Carbamidomethyl(C)@2 |
|  |  |  |  |  |  |  |  | 99.00 | AQNAHAYVNAAFLLK |  |
|  |  |  |  |  |  |  |  | 99.00 | FLQNSTNATGVLR |  |
|  |  |  |  |  |  |  |  | 99.00 | HIDLIANVPVR |  |
|  |  |  |  |  |  |  |  | 99.00 | LAGNSVEHFTLN |  |
|  |  |  |  |  |  |  |  | 99.00 | NLFENAVVQEALK |  |
|  |  |  |  |  |  |  |  | 95.00 | TVPVDTSLNTFIR |  |
|  |  |  |  |  |  |  |  | 90.00 | SLAYDAEPR |  |
| **0015** | 13.87 | AAEL010382-PA | Q16T47_AEDAE | 139,591 | 6,28 | aldehyde oxidase | 8 | 99.00 | FLQNSTNATGVLR |  |
|  |  |  |  |  |  |  |  | 99.00 | HIDLIANVPVR |  |
|  |  |  |  |  |  |  |  | 99.00 | MIELMPEFR |  |
|  |  |  |  |  |  |  |  | 99.00 | TWTYKPPGAK |  |
|  |  |  |  |  |  |  |  | 98.00 | ACEIILER | Carbamidomethyl(C)@2 |
|  |  |  |  |  |  |  |  | 98.00 | LAGNSVEHFTLN |  |
|  |  |  |  |  |  |  |  | 96.00 | SLAYDAEPR |  |
|  |  |  |  |  |  |  |  | 89.00 | AQNAHAYVNAAFLLK |  |
| **0017** | 6.73 | AAEL010382-PA | Q16T47_AEDAE | 139,591 | 6,28 | aldehyde oxidase | 4 | 99.00 | HIDLIANVPVR |  |
|  |  |  |  |  |  |  |  | 99.00 | NLFENAVVQEALK |  |
|  |  |  |  |  |  |  |  | 96.00 | FLQNSTNATGVLR |  |
|  |  |  |  |  |  |  |  | 95.00 | TVPVDTSLNTFIR |  |
| **0018** | 6.36 | AAEL009691-PA | Q16V52_AEDAE | 130,634 | 6,43 | carboxylase:pyruvate/acetyl-coa/propionyl-coa | 4 | 99.00 | SDFAQAVIDAGLR |  |
|  |  |  |  |  |  |  |  | 99.00 | TNIPFLLNVLENQK |  |
|  |  |  |  |  |  |  |  | 98.00 | VGEEFEVTIEK |  |
|  |  |  |  |  |  |  |  | 78.00 | EVFFELNGQLR |  |
| **0020** | 8 | AAEL010382-PA | Q16T47_AEDAE | 139,591 | 6,28 | aldehyde oxidase | 4 | 99.00 | FLQNSTNATGVLR |  |
|  |  |  |  |  |  |  |  | 99.00 | HIDLIANVPVR |  |
|  |  |  |  |  |  |  |  | 99.00 | LQVFVDVSSIEELR |  |
|  |  |  |  |  |  |  |  | 99.00 | NLFENAVVQEALK |  |
| **0021** | 13.22 | AAEL009185-PA | Q1HR67_AEDAE | 39,874 | 5,97 | arginine or creatine kinase | 7 | 99.00 | EMYDGISELIK | Oxidation(M)@2 |
|  |  |  |  |  |  |  |  | 99.00 | FLQAANACR | Carbamidomethyl(C)@8 |
|  |  |  |  |  |  |  |  | 99.00 | FYPLTGMDK | Oxidation(M)@7 |
|  |  |  |  |  |  |  |  | 99.00 | LESIADKYNLQVR |  |
|  |  |  |  |  |  |  |  | 99.00 | MGLTEYQAVK | Oxidation(M)@1 |
|  |  |  |  |  |  |  |  | 99.00 | RLVTAVNDIEKR |  |
|  |  |  |  |  |  |  |  | 94.00 | LVTAVNDIEKR |  |
|  | 2.55 | AAEL006693-PB | Q175D6_AEDAE | 40,006 | 5,66 | uroporphyrinogen decarboxylase | 3 | 99.00 | YLPEFQEVR |  |
|  | 0 | AAEL006693-PA | Q175D5_AEDAE | 46,392 | 5,29 | uroporphyrinogen decarboxylase |  | 72.00 | EFPPLKNDNLLR |  |
|  | 0 | AAEL006693-PC | Q175D4_AEDAE | 34,486 | 5,52 | uroporphyrinogen decarboxylase |  |  |  |  |
| **0023** | 6 | AAEL009691-PA | Q16V52_AEDAE | 130,634 | 6,43 | carboxylase:pyruvate/acetyl-coa/propionyl-coa | 3 | 99.00 | EVFFELNGQLR |  |
|  |  |  |  |  |  |  |  | 99.00 | GLAPVEAYLSIPEIIR |  |
|  |  |  |  |  |  |  |  | 99.00 | TNIPFLLNVLENQK |  |
| **0033** | 6.01 | AAEL009691-PA | Q16V52_AEDAE | 130,634 | 6,43 | carboxylase:pyruvate/acetyl-coa/propionyl-coa | 3 | 99.00 | EVFFELNGQLR |  |
|  |  |  |  |  |  |  |  | 99.00 | SDFAQAVIDAGLR |  |
|  |  |  |  |  |  |  |  | 99.00 | TNIPFLLNVLENQK |  |
| **0075** | 2.43 | AAEL012278-PA | Q16MK3_AEDAE | 115,377 | 5,98 | metalloprotease | 2 | 99.00 | FPVRDPFFK |  |
|  | 0 | AAEL007254-PA | Q172U8_AEDAE | 97,068 | 6,11 | metalloprotease |  | 63.00 | LGVLQQLDVPIAPIDR |  |
| **0076** | 2.7 | AAEL012278-PA | Q16MK3_AEDAE | 115,377 | 5,98 | metalloprotease | 2 | 99.00 | LGVLQQLDVPIAPIDR |  |
|  |  |  |  |  |  |  |  | 80.00 | DSNNVFSINFR |  |
| **0087** | 7.53 | AAEL007201-PA | Q173A7_AEDAE | 113,755 | 5,88 | glutamyl aminopeptidase | 4 | 99.00 | TGLLNDAFALADASQLR |  |
|  |  |  |  |  |  |  |  | 99.00 | VYTTPFQIQNAR |  |
|  |  |  |  |  |  |  |  | 99.00 | YDLALELTR |  |
|  |  |  |  |  |  |  |  | 97.00 | TVVYYYGLQR |  |
| **0093** | 4 | AAEL005429-PA | Q17A22_AEDAE | 101,917 | 6,23 | 2-oxoglutarate dehydrogenase | 2 | 99.00 | GVFGFIPKPSK |  |
|  |  |  |  |  |  |  |  | 99.00 | SQAFDNFLATKFPTVK | Deamidated(N)@6 |
| **0094** | 5.46 | AAEL008216-PA | Q16ZG5_AEDAE | 98,744 | 5,8 | aconitase | 4 | 99.00 | FDTEVDLEYYR |  |
|  |  |  |  |  |  |  |  | 99.00 | TDTELEIPFKPAR |  |
|  |  |  |  |  |  |  |  | 91.00 | AGTNPFQNLQK | Protein Terminal Acetyl@N-term |
|  |  |  |  |  |  |  |  | 61.00 | FVEFFGPGVSELSIADR |  |
| **0095** | 4 | AAEL008216-PA | Q16ZG5_AEDAE | 98,744 | 5,8 | aconitase | 2 | 99.00 | FVEFFGPGVSELSIADR |  |
|  |  |  |  |  |  |  |  | 99.00 | TDTELEIPFKPAR |  |
| **0096** | 13.7 | AAEL005429-PA | Q17A22_AEDAE | 101,917 | 6,23 | 2-oxoglutarate dehydrogenase | 7 | 99.00 | DVFIDLNCFR | Carbamidomethyl(C)@8 |
|  |  |  |  |  |  |  |  | 99.00 | GLPEFPADAK |  |
|  |  |  |  |  |  |  |  | 99.00 | GVFGFIPKPSK |  |
|  |  |  |  |  |  |  |  | 99.00 | HYYNLNNER |  |
|  |  |  |  |  |  |  |  | 99.00 | LESLCPFPTQR | Carbamidomethyl(C)@5 |
|  |  |  |  |  |  |  |  | 99.00 | SQAFDNFLATKFPTVK |  |
|  |  |  |  |  |  |  |  | 98.00 | LNVLTTLFQTRPVK |  |
| **0108** | 8.44 | AAEL012918-PA | Q16KP9_AEDAE | 98,005 | 5,43 | puromycin-sensitive aminopeptidase | 5 | 99.00 | ALGSISDVQILR |  |
|  |  |  |  |  |  |  |  | 99.00 | ETFVLVDSENTSLIR |  |
|  |  |  |  |  |  |  |  | 99.00 | SKYFTPSGEER |  |
|  |  |  |  |  |  |  |  | 97.00 | FAGVTQFEATDAR |  |
|  |  |  |  |  |  |  |  | 88.00 | YEGGFLLSR |  |
| **0112** | 8.64 | AAEL007201-PA | Q173A7_AEDAE | 113,755 | 5,88 | glutamyl aminopeptidase | 5 | 99.00 | GWLDSDVAVHPDLR |  |
|  |  |  |  |  |  |  |  | 99,00 | HGEQIAWDHVR |  |
|  |  |  |  |  |  |  |  | 99,00 | TGLLNDAFALADASQLR |  |
|  |  |  |  |  |  |  |  | 99.00 | YDLALELTR |  |
|  |  |  |  |  |  |  |  | 77.00 | TVVYYYGLQR |  |
| **0130** | 6.02 | AAEL008216-PA | Q16ZG5_AEDAE | 98,744 | 5,8 | aconitase | 3 | 99.00 | ADDKVQVIEAYLK |  |
|  |  |  |  |  |  |  |  | 99.00 | FVEFFGPGVSELSIADR |  |
|  |  |  |  |  |  |  |  | 99.00 | TDTELEIPFKPAR |  |
| **0146** | 5.92 | AAEL002870-PB | Q17GV2_AEDAE | 81,878 | 5,08 | dipeptidyl peptidase iii | 5 | 99.00 | LIFVQANTELNADGAVQLK |  |
|  | 0 | AAEL002870-PA |  |  |  | dipeptidyl peptidase iii |  | 98.00 | KSIFLLTER |  |
|  |  |  |  |  |  |  |  | 93.00 | IVPNLEEDKLELIVK |  |
|  |  |  |  |  |  |  |  | 76.00 | AEAVGLYLSLNR |  |
|  |  |  |  |  |  |  |  | 64.00 | GEFEGFVAMVNK | Oxidation(M)@9 |
| **0163** | 4 | AAEL008216-PA | Q16ZG5_AEDAE | 98,744 | 5,8 | aconitase | 2 | 99.00 | FVEFFGPGVSELSIADR |  |
|  |  |  |  |  |  |  |  | 99.00 | TDTELEIPFKPAR |  |
|  | 3.34 | AAEL007915-PA | MOEH_AEDAE | 69,067 | 5,75 | moesin/ezrin/radixin | 3 | 99.00 | APDFVFFAPR |  |
|  |  |  |  |  |  |  |  | 81.00 | QKLEDEIR |  |
|  |  |  |  |  |  |  |  | 76.00 | KAPDFVFFAPR |  |
| **0195** | 1.72 | AAEL004369-PA | Q17D16_AEDAE | 73,575 | 5,63 | alpha-glucosidase | 2 | 94.00 | DLDTAEFSLFTIAR |  |
|  |  |  |  |  |  |  |  | 68,00 | SAHLSQDLPIFVR |  |
| **0199** | 2.76 | AAEL015458-PA | Q1DGV7_AEDAE | 70,561 | 7,71 | transferrin | 2 | 99,00 | LQQFYQDAK |  |
|  | 0 | AAEL015639-PA | Q1DGG7_AEDAE | 50,307 | 7,79 | transferrin |  | 72,00 | EVPVQPGDHLNR |  |
| **0201** | 10 | AAEL004580-PA | Q17CH4_AEDAE | 73,973 | 4,87 | beta-galactosidase | 5 | 99,00 | AQVYVDEFYVGTLSR |  |
|  |  |  |  |  |  |  |  | 99,00 | DPSQLTINDLR |  |
|  |  |  |  |  |  |  |  | 99,00 | ELLNEGSNEIILVEQQR |  |
|  |  |  |  |  |  |  |  | 99,00 | LSILVENQGR |  |
|  |  |  |  |  |  |  |  | 99,00 | NIIGEYIELPAVPVPDR |  |
| **0234** | 15.15 | AAEL004434-PA | Q17CT0_AEDAE | 67,933 | 654 | transketolase | 8 | 99,00 | HLGVEQVPR |  |
|  |  |  |  |  |  |  |  | 99,00 | KIDSDLEGHPTPR |  |
|  |  |  |  |  |  |  |  | 99,00 | KVNIGNVQLATPPAYQLGESVATR |  |
|  |  |  |  |  |  |  |  | 99,00 | LDNLCVIFDVNR | Carbamidomethyl(C)@5 |
|  |  |  |  |  |  |  |  | 99,00 | QGGIGEAVLSAVADQR | Gln->pyro-Glu@N-term |
|  |  |  |  |  |  |  |  | 99,00 | TIAFVSTFATFFTR |  |
|  |  |  |  |  |  |  |  | 99,00 | TIPGSTVFYPSDAVSTER |  |
|  |  |  |  |  |  |  |  | 93,00 | TSRPNTAVLYENNEPFQVGK |  |
| **0236** | 8 | AAEL004434-PA | Q17CT0_AEDAE | 67,933 | 654 | transketolase | 4 | 99,00 | HLGVEQVPR |  |
|  |  |  |  |  |  |  |  | 99,00 | KVNIGNVQLATPPAYQLGESVATR |  |
|  |  |  |  |  |  |  |  | 99,00 | TIAFVSTFATFFTR |  |
|  |  |  |  |  |  |  |  | 99,00 | TIPGSTVFYPSDAVSTER |  |
| **0243** | 10.6 | AAEL011778-PA | Q16P29_AEDAE | 65,799 | 6,11 | wd-repeat protein | 7 | 99,00 | ASGDFVYTAGIDDSIK |  |
|  |  |  |  |  |  |  |  | 99,00 | IITGSEDNTIGVFEGPPFK |  |
|  |  |  |  |  |  |  |  | 99,00 | QISIEGNTYTGVDAK |  |
|  |  |  |  |  |  |  |  | 99,00 | VGGVGHGNQINDIR |  |
|  |  |  |  |  |  |  |  | 98,00 | NEFQPIGGPIK |  |
|  |  |  |  |  |  |  |  | 67,00 | YSPSGHLFASAGFDGK |  |
|  |  |  |  |  |  |  |  | 46,00 | NFLYTNGHSVIIR | Deamidated(N)@6 |
| **0252** | 16 | AAEL011584-PA | Q16PM9_AEDAE | 60,794 | 5,47 | chaperonin-60kD, ch60 | 8 | 99,00 | AAVEEGIVPGGGTALLR |  |
|  |  |  |  |  |  |  |  | 99,00 | AVTSPEEIAQVATISANGDR |  |
|  |  |  |  |  |  |  |  | 99,00 | GYISPYFINSSK |  |
|  |  |  |  |  |  |  |  | 99,00 | ISSVQSIIPALELANSAR |  |
|  |  |  |  |  |  |  |  | 99,00 | KISSVQSIIPALELANSAR |  |
|  |  |  |  |  |  |  |  | 99,00 | KPLVIIAEDVDGEALSTLVVNR |  |
|  |  |  |  |  |  |  |  | 99,00 | LVQDVANNTNEEAGDGTTTATVLAR |  |
|  |  |  |  |  |  |  |  | 99,00 | VEFQDALVLFSEK |  |
| **0254** | 20.21 | AAEL011584-PA | Q16PM9_AEDAE | 60,794 | 5,47 | chaperonin-60kD, ch60 | 10 | 99,00 | AAVEEGIVPGGGTALLR |  |
|  |  |  |  |  |  |  |  | 99,00 | AIGDLISEAMKR | Dethiomethyl(M)@10 |
|  |  |  |  |  |  |  |  | 99,00 | AVTSPEEIAQVATISANGDR |  |
|  |  |  |  |  |  |  |  | 99,00 | GANPVEIRR |  |
|  |  |  |  |  |  |  |  | 99,00 | GYISPYFINSSK |  |
|  |  |  |  |  |  |  |  | 99,00 | ISSVQSIIPALELANSAR |  |
|  |  |  |  |  |  |  |  | 99,00 | KISSVQSIIPALELANSAR |  |
|  |  |  |  |  |  |  |  | 99,00 | KPLVIIAEDVDGEALSTLVVNR |  |
|  |  |  |  |  |  |  |  | 99,00 | LVQDVANNTNEEAGDGTTTATVLAR |  |
|  |  |  |  |  |  |  |  | 99,00 | VEFQDALVLFSEK |  |
| **0259** | 20 | AAEL011584-PA | Q16PM9_AEDAE | 60,794 | 5,47 | chaperonin-60kD, ch60 | 10 | 99,00 | AAVEEGIVPGGGTALLR |  |
|  |  |  |  |  |  |  |  | 99,00 | AIGDLISEAMKR |  |
|  |  |  |  |  |  |  |  | 99,00 | ALHQPCTQIAK | Carbamidomethyl(C)@6 |
|  |  |  |  |  |  |  |  | 99,00 | AVTSPEEIAQVATISANGDR | Deamidated(N)@17 |
|  |  |  |  |  |  |  |  | 99,00 | GANPVEIRR |  |
|  |  |  |  |  |  |  |  | 99,00 | ISSVQSIIPALELANSAR |  |
|  |  |  |  |  |  |  |  | 99,00 | KISSVQSIIPALELANSAR |  |
|  |  |  |  |  |  |  |  | 99,00 | KPLVIIAEDVDGEALSTLVVNR |  |
|  |  |  |  |  |  |  |  | 99,00 | LVQDVANNTNEEAGDGTTTATVLAR | Oxidation(N)@7; Dehydrated(T)@9; Deamidated(N)@10 |
|  |  |  |  |  |  |  |  | 99,00 | NVILEQSWGSPK |  |
| **0267** | 11.52 | AAEL005790-PA | Q178T5_AEDAE | 72,184 | 6,06 | malic enzyme | 7 | 99,00 | GLAFTIEER |  |
|  |  |  |  |  |  |  |  | 99,00 | LAEIVTEDDLER |  |
|  |  |  |  |  |  |  |  | 99,00 | LGQWPVESDSEVAGGVSGFGR |  |
|  |  |  |  |  |  |  |  | 99,00 | NCLIQFEDFGNSNAFR | Carbamidomethyl(C)@2 |
|  |  |  |  |  |  |  |  | 99,00 | QALGLHGLLPATVR | Gln->pyro-Glu@N-term |
|  |  |  |  |  |  |  |  | 97,00 | NWPETDVR |  |
|  |  |  |  |  |  |  |  | 99,00 | QALGLHGLLPATVR |  |
| **0283** | 11,52 | AAEL002886-PB | Q17GT5_AEDAE | 54,019 | 6,21 | thioredoxin reductase | 7 | 99,00 | NTVGIHPTVAEEFTR |  |
|  | 0 | AAEL002886-PA | Q17GT4_AEDAE | 56,707 | 7,17 | thioredoxin reductase |  | 99,00 | NVVIAVGGRPR |  |
|  |  |  |  |  |  |  |  | 99,00 | SDDGTEGSDVYDTVLFAIGR |  |
|  |  |  |  |  |  |  |  | 99,00 | VAVLDFVKPSPR |  |
|  |  |  |  |  |  |  |  | 99,00 | YRSDDGTEGSDVYDTVLFAIGR |  |
|  |  |  |  |  |  |  |  | 97,00 | VEYVNGLGYFK |  |
|  |  |  |  |  |  |  |  | 97,00 | VAVLDFVKPSPR | Carbamidomethyl@N-term |
| **0290** | 5.45 | AAEL013407-PA | Q16J86_AEDAE | 56,899 | 7,71 | catalase | 3 | 99,00 | ADELAGSDPDYSIR | Deoxy(D)@10 |
|  |  |  |  |  |  |  |  | 99,00 | NPAENQLNLFK |  |
|  |  |  |  |  |  |  |  | 96,00 | FSTVGGESGSADTAR |  |
| **0302** | 14.57 | AAEL000641-PB | Q1HR78_AEDAE | 55,954 | 4,95 | protein disulfide isomerase | 9 | 99,00 | AVFDGEYTEEALKK |  |
|  | 0 | AAEL000641-PA |  |  |  | protein disulfide isomerase |  | 99,00 | ILEFFGMK | Oxidation(M)@7 |
|  |  |  |  |  |  |  |  | 99,00 | ILEFFGMKK | Oxidation(M)@7 |
|  |  |  |  |  |  |  |  | 99,00 | ILFVTIDADQEDHQR |  |
|  |  |  |  |  |  |  |  | 99,00 | INSFPTIYLYR |  |
|  |  |  |  |  |  |  |  | 99,00 | NGTPIEYTGGR |  |
|  |  |  |  |  |  |  |  | 99,00 | TLEGFVNFLEGK |  |
|  |  |  |  |  |  |  |  | 50,00 | YKPETNDLAADKVELFVSK |  |
|  |  |  |  |  |  |  |  | 46,00 | VLVADKFDEVAMDSTK | Oxidation(M)@12 |
| **0304** | 14 | AAEL006823-PA | Q174Q7_AEDAE | 59,651 | 5,46 | amp dependent ligase | 7 | 99,00 | GVAVSQAHIAVVLGRPVK |  |
|  |  |  |  |  |  |  |  | 99,00 | HPAPVLDPEANLGR |  |
|  |  |  |  |  |  |  |  | 99,00 | LILSILDRNPEK |  |
|  |  |  |  |  |  |  |  | 99,00 | SGFNEDVFYDVLER |  |
|  |  |  |  |  |  |  |  | 99,00 | TGDIGYLDEEGFVYLVDR |  |
|  |  |  |  |  |  |  |  | 99,00 | TSLPFLGYYNDEEANR |  |
|  |  |  |  |  |  |  |  | 99,00 | VAQNLTALGFR |  |
| **0305** | 14 | AAEL006823-PA | Q174Q7_AEDAE | 59,651 | 5,46 | amp dependent ligase | 7 | 99,00 | GVAVSQAHIAVVLGRPVK |  |
|  |  |  |  |  |  |  |  | 99,00 | HPAPVLDPEANLGR |  |
|  |  |  |  |  |  |  |  | 99,00 | LILSILDRNPEK |  |
|  |  |  |  |  |  |  |  | 99,00 | SGFNEDVFYDVLER |  |
|  |  |  |  |  |  |  |  | 99,00 | TGDIGYLDEEGFVYLVDR |  |
|  |  |  |  |  |  |  |  | 99,00 | TSLPFLGYYNDEEANR |  |
|  |  |  |  |  |  |  |  | 99,00 | VAQNLTALGFR |  |
| **0319** | 8 | AAEL011309-PA | Q16QF3_AEDAE | 53,279 | 5,87 | orotidine-5'-phosphate decarboxylase, putative | 4 | 99,00 | FADIGNTVALQYSSGPFK |  |
|  |  |  |  |  |  |  |  | 99,00 | KFADIGNTVALQYSSGPFK |  |
|  |  |  |  |  |  |  |  | 99,00 | SEGLVITDAIVVVDR |  |
|  |  |  |  |  |  |  |  | 99,00 | VGINSPVYFDLR |  |
|  | 4.22 | AAEL011137-PA | Q16QY3_AEDAE | 49,553 | 6,17 | succinyl-coa:3-ketoacid-coenzyme a transferase | 2 | 99,00 | AVFDVDQER |  |
|  |  |  |  |  |  |  |  | 99,00 | QYLTGELELELTPQGTLAER |  |
| **0345** | 13.52 | AAEL009387-PB | Q16VW5_AEDAE | 51,552 | 5,64 | hexokinase | 7 | 99,00 | AGLLFGGVGSDILFK |  |
|  | 0 | AAEL009387-PA | Q16VW4_AEDAE | 50,499 | 5,75 | hexokinase |  | 99,00 | EIDHFSINPGR |  |
|  |  |  |  |  |  |  |  | 99,00 | FLALDLGGTNFR |  |
|  |  |  |  |  |  |  |  | 99,00 | GAALVAAVACR | Carbamidomethyl(C)@10 |
|  |  |  |  |  |  |  |  | 99,00 | GKFLALDLGGTNFR |  |
|  |  |  |  |  |  |  |  | 99,00 | QFVKPDISFDLMLSEDGSGR | Oxidation(M)@12 |
|  |  |  |  |  |  |  |  | 97,00 | SVTVGVDGSVYR |  |
| **0358** | 8.31 | AAEL003746-PA | Q0IG02_AEDAE | 51,849 | 8,15 | 4-Hydroxybutyrate CoA-transferase, putative | 5 | 99,00 | GKPIIALPSITNK |  |
|  |  |  |  |  |  |  |  | 99,00 | IVGSFLIGTK |  |
|  |  |  |  |  |  |  |  | 99,00 | KPTYTTAEEAVK |  |
|  |  |  |  |  |  |  |  | 97,00 | YYTYSNQISQPLER |  |
|  |  |  |  |  |  |  |  | 83,00 | IVGSFLIGTKK |  |
| **0360** | 10.17 | AAEL003746-PA | Q0IG02_AEDAE | 51,894 | 8,15 | 4-Hydroxybutyrate CoA-transferase, putative | 5 | 99,00 | AYELIQIAHPDHR |  |
|  |  |  |  |  |  |  |  | 99,00 | GKPIIALPSITNK |  |
|  |  |  |  |  |  |  |  | 99,00 | IVGSFLIGTK |  |
|  |  |  |  |  |  |  |  | 99,00 | IVGSFLIGTKK |  |
|  |  |  |  |  |  |  |  | 99,00 | KPTYTTAEEAVK |  |
| **0364** | 6.42 | AAEL001668-PA | Q17KK5_AEDAE | 46,621 | 6,28 | enolase | 4 | 99,00 | GNPTVEVDLVTDLGLFR |  |
|  |  |  |  |  |  |  |  | 99,00 | IGSEVYHHLK |  |
|  |  |  |  |  |  |  |  | 98,00 | FGLDATAVGDEGGFAPNILENK |  |
|  |  |  |  |  |  |  |  | 63,00 | KNGWGTMVSHR | Deamidated(N)@2 |
| **0375** | 5.15 | AAEL009872-PA | Q16UK8_AEDAE | 59,521 | 8,6 | alanine aminotransferase | 3 | 99,00 | DGGVPSDPNNIILSAGASGGIK |  |
|  |  |  |  |  |  |  |  | 99,00 | ILVVINPGNPTGQVLSR |  |
|  |  |  |  |  |  |  |  | 93,00 | QVLGLVSYPPLFEDK |  |
| **0424** | 4.61 | AAEL012579-PA | Q16LN3_AEDAE | 45,363 | 7,07 | aspartate aminotransferase | 3 | 99,00 | AAVASQITLLIR |  |
|  |  |  |  |  |  |  |  | 99,00 | IVNLVLNDATLR |  |
|  |  |  |  |  |  |  |  | 62,00 | NFGLYNER |  |
|  | 4 | AAEL002160-PA | Q17J37_AEDAE | 44,79 | 6,59 | gtp-binding protein | 2 | 99,00 | DLAIIAEELR |  |
|  |  |  |  |  |  |  |  | 99,00 | ILEPEVKPLIGR |  |
| **0433** | 14.74 | AAEL000641-PB | Q1HR78_AEDAE | 55,954 | 4,95 | protein disulfide isomerase | 8 | 99,00 | AVFDGEYTEEALKK |  |
|  | 0 | AAEL000641-PA |  |  |  | protein disulfide isomerase |  | 99,00 | ELETVAEAEEFLK |  |
|  |  |  |  |  |  |  |  | 99,00 | IIHLEEDMAK | Oxidation(M)@8 |
|  |  |  |  |  |  |  |  | 99,00 | ILEFFGMK | Oxidation(M)@7 |
|  |  |  |  |  |  |  |  | 99,00 | ILEFFGMKK | Oxidation(M)@7 |
|  |  |  |  |  |  |  |  | 99,00 | ILFVTIDADQEDHQR |  |
|  |  |  |  |  |  |  |  | 99,00 | NGTPIEYTGGR | Deamidated(N)@1 |
|  |  |  |  |  |  |  |  | 80,00 | YKPETNDLAADKVELFVSK |  |
|  | 7.7 | AAEL011197-PB | Q16QR7_AEDAE | 41,822 | 5,3 | actin | 4 | 99,00 | GYSFTTTAER |  |
|  | 0 | AAEL011197-PA |  |  |  | actin |  | 99,00 | SYELPDGQVITIGNER |  |
|  | 0 | AAEL005961-PA | Q178A9_AEDAE | 41,643 | 5,29 | actin |  | 99,00 | VAPEEHPVLLTEAPLNPK |  |
|  | 0 | AAEL004631-PA | Q17C87_AEDAE | 41,788 | 5,29 | actin |  | 98,00 | AVFPSIVGRPR |  |
|  | 0 | AAEL001951-PA | Q6QNY2_AEDAE | 41,582 | 5,29 | actin |  |  |  |  |
|  | 0 | AAEL001928-PA | ACT1_AEDAE | 41,673 | 5,3 | actin |  |  |  |  |
|  | 0 | AAEL001673-PA | Q17KG3_AEDAE | 41,777 | 5,22 | actin |  |  |  |  |
|  | 0 | AAEL009451-PA | Q16VS2_AEDAE | 41,576 | 5,36 | actin |  |  |  |  |
|  | 0 | AAEL005964-PA | Q178B0_AEDAE | 41,546 | 5,29 | actin |  |  |  |  |
| **0442** | 4 | AAEL001887-PB | Q17JX2_AEDAE | 44,672 | 6,62 | glutamine synthetase 1, 2 (glutamate-amonia ligase) (gs) | 2 | 99,00 | LETSSIDKFSWGVADR |  |
|  | 0 | AAEL001887-PA |  |  |  | glutamine synthetase 1, 2 (glutamate-amonia ligase) (gs) |  | 99,00 | YVQATYVWIDGTGENVR | Dioxidation(W)@8 |
| **0445** | 4.27 | AAEL001887-PB | Q17JX2_AEDAE | 44,672 | 6,62 | glutamine synthetase 1, 2 (glutamate-amonia ligase) (gs) | 3 | 99,00 | ILQESPNAYMNK | Oxidation(M)@10 |
|  | 0 | AAEL001887-PA |  |  |  | glutamine synthetase 1, 2 (glutamate-amonia ligase) (gs) |  | 99,00 | YVQATYVWIDGTGENVR |  |
|  |  |  |  |  |  |  |  | 46,00 | LETSSIDKFSWGVADR |  |
| **0447** | 15.52 | AAEL005766-PB | Q178U8_AEDAE | 39,484 | 8,45 | fructose-bisphosphate aldolase | 8 | 99,00 | AAQDELIKR |  |
|  | 0 | AAEL005766-PA | Q178U9_AEDAE | 39,12 | 8,02 | fructose-bisphosphate aldolase |  | 99,00 | GILAADESTATCGKR | Carbamidomethyl(C)@12 |
|  |  |  |  |  |  |  |  | 99,00 | IVPIVEPEILPDGDHDLER |  |
|  |  |  |  |  |  |  |  | 99,00 | KPSAQEIALATVLALR |  |
|  |  |  |  |  |  |  |  | 99,00 | NTPSYQAILENANVLAR |  |
|  |  |  |  |  |  |  |  | 99,00 | TTYFNYPPKDVQEELAR | Protein Terminal Acetyl@N-term |
|  |  |  |  |  |  |  |  | 99,00 | YASVCQSQR | Carbamidomethyl(C)@5 |
|  |  |  |  |  |  |  |  | 97,00 | FADIGVENNEDNRR |  |
| **0467** | 6.03 | AAEL007555-PA | Q171S4_AEDAE | 46,41 | 6,05 | acyl-coa dehydrogenase | 4 | 99,00 | LAQEQIAPLVR |  |
|  |  |  |  |  |  |  |  | 99,00 | VPEENILGEFGK |  |
|  |  |  |  |  |  |  |  | 97,00 | GYQYAAGFLNEGR |  |
|  |  |  |  |  |  |  |  | 69,00 | LYASEVAQR |  |
| **0469** | 13.22 | AAEL009185-PA | Q1HR67_AEDAE | 39,874 | 5,97 | arginine or creatine kinase | 7 | 99,00 | EMYDGISELIK | Oxidation(M)@2 |
|  |  |  |  |  |  |  |  | 99,00 | FLQAANACR | Carbamidomethyl(C)@8 |
|  |  |  |  |  |  |  |  | 99,00 | FYPLTGMDK | Oxidation(M)@7 |
|  |  |  |  |  |  |  |  | 99,00 | LESIADKYNLQVR |  |
|  |  |  |  |  |  |  |  | 99,00 | MGLTEYQAVK | Oxidation(M)@1 |
|  |  |  |  |  |  |  |  | 99,00 | RLVTAVNDIEKR |  |
|  |  |  |  |  |  |  |  | 94,00 | LVTAVNDIEKR |  |
|  | 2.55 | AAEL006693-PB | Q175D6_AEDAE | 40,006 | 5,66 | uroporphyrinogen decarboxylase | 2 | 99,00 | YLPEFQEVR |  |
|  | 0 | AAEL006693-PA | Q175D5_AEDAE | 46,392 | 5,29 | uroporphyrinogen decarboxylase |  | 72,00 | EFPPLKNDNLLR |  |
|  | 0 | AAEL006693-PC | Q175D4_AEDAE | 34,486 | 5,52 | uroporphyrinogen decarboxylase |  |  |  |  |
| **0470** | 7.61 | AAEL007555-PA | Q171S4_AEDAE | 46,41 | 6,05 | acyl-coa dehydrogenase | 4 | 99,00 | GYQYAAGFLNEGR |  |
|  |  |  |  |  |  |  |  | 99,00 | LYASEVAQR |  |
|  |  |  |  |  |  |  |  | 99,00 | VPEENILGEFGK |  |
|  |  |  |  |  |  |  |  | 96,00 | LAQEQIAPLVR |  |
| **0477** | 2.7 | AAEL015034-PA | Q16ET2_AEDAE | 36,546 | 5,64 | alcohol dehydrogenase | 2 | 99,00 | SLGFDHAINYK |  |
|  |  |  |  |  |  |  |  | 80,00 | AFSGLPTADNFR |  |
| **0501** | 6 | AAEL008006-PA | Q0IEU5_AEDAE | 35,069 | 5,93 | 3-hydroxyacyl-coa dehyrogenase | 3 | 99,00 | ALQLTQDELNSLER |  |
|  |  |  |  |  |  |  |  | 99,00 | EIEGFALNR |  |
|  |  |  |  |  |  |  |  | 99,00 | IGIIGSGLIGR |  |
|  | 4 | AAEL004086-PB | Q17DN2_AEDAE | 37,076 | 5,51 | aldo-keto reductase | 2 | 99,00 | SIGVSNFNSEQITR |  |
|  | 0 | AAEL004086-PA | Q17DN1_AEDAE | 32,31 | 5,47 | aldo-keto reductase |  | 99,00 | YLVDIGTIPIPK |  |
| **0539** | 10 | AAEL001022-PB | Q17MF0_AEDAE | 36,756 | 5,48 | anterior fat body protein | 5 | 99,00 | FNEGKVDPK |  |
|  | 0 | AAEL001022-PA | Q17ME9_AEDAE | 34,132 | 5,25 | anterior fat body protein |  | 99,00 | KVELEIQIPAK |  |
|  |  |  |  |  |  |  |  | 99,00 | LLLVSWDGR |  |
|  |  |  |  |  |  |  |  | 99,00 | LYAGTMQLESLGDIFQQK | Oxidation(M)@6 |
|  |  |  |  |  |  |  |  | 99,00 | LYYIDTAALDIK |  |
| **0554** | 10 | AAEL011302-PE | Q1HR06_AEDAE | 35,622 | 4,67 | annexin | 5 | 99,00 | EAFQEMYGK | Oxidation(M)@6 |
|  | 0 | AAEL011302-PD | Q16QE7_AEDAE | 35,726 | 4,83 | annexin |  | 99,00 | EFSGAIEEGFK |  |
|  | 0 | AAEL011302-PC | Q16QE9_AEDAE | 35,641 | 4,64 | annexin |  | 99,00 | PFDANEDAATLR |  |
|  | 0 | AAEL011302-PB | Q16QE8_AEDAE | 35,738 | 4,71 | annexin |  | 99,00 | SEIDLGDIKEAFQEMYGK | Oxidation(M)@15 |
|  | 0 | AAEL011302-PA | Q16QF1_AEDAE | 35,756 | 4,7 | annexin |  | 99,00 | TIAEFYEQLYGVSLESDLKGDTSGAFKR | Deamidated(Q)@8; Oxidation(Y)@10; Dehydrated(S)@13 |
| **0568** | 12.66 | AAEL013739-PA | Q16IA8_AEDAE | 34,404 | 8,43 | electron transport oxidoreductase | 7 | 99,00 | AAVDAGFVPNDLQIGQTGK |  |
|  |  |  |  |  |  |  |  | 99,00 | GTNFEAAGTGGSAAVEKAPEGNYK |  |
|  |  |  |  |  |  |  |  | 99,00 | KLGGDVTVLVAGTK |  |
|  |  |  |  |  |  |  |  | 99,00 | LDVSPVSEIIDVK |  |
|  |  |  |  |  |  |  |  | 99,00 | SDLTEFVSQELTK |  |
|  |  |  |  |  |  |  |  | 99,00 | TIYAGNAIQTVK |  |
|  |  |  |  |  |  |  |  | 78,00 | SDRPSLTAAK |  |
|  | 4 | AAEL000219-PA | Q17PU4_AEDAE | 32,426 | 5,84 | lactoylglutathione lyase | 2 | 99,00 | IAFAVPFDVQPK |  |
|  |  |  |  |  |  |  |  | 99,00 | VSLNVTDLER |  |
| **0569** | 9.4 | AAEL004930-PA | Q17BL7_AEDAE | 31,307 | 5,73 | carbonic anhydrase | 5 | 99,00 | EPIEVSHDQLELFR |  |
|  |  |  |  |  |  |  |  | 99,00 | SLVNPGYCWR | Carbamidomethyl(C)@8 |
|  |  |  |  |  |  |  |  | 99,00 | VVNNYRPPLELGNR |  |
|  |  |  |  |  |  |  |  | 99,00 | WTYIPENTR |  |
|  |  |  |  |  |  |  |  | 96,00 | VGRPHPELDTIAK |  |
|  | 8 | AAEL000219-PA | Q17PU4_AEDAE | 32,426 | 5,84 | lactoylglutathione lyase | 4 | 99,00 | DILGMQVLR |  |
|  |  |  |  |  |  |  |  | 99,00 | IAFAVPFDVQPK |  |
|  |  |  |  |  |  |  |  | 99,00 | VSLNVTDLER |  |
|  |  |  |  |  |  |  |  | 99,00 | YWHGTLEMK |  |
| **0571** | 9.71 | AAEL003957-PC | Q1HQF5_AEDAE | 17,105 | 6,74 | conserved hypothetical protein | 7 | 99,00 | MLYSSSFDALKK | Oxidation(M)@1 |
|  | 0 | AAEL003957-PB |  |  |  | conserved hypothetical protein |  | 99,00 | NAEYDQFLEDIQK |  |
|  | 0 | AAEL003957-PA |  |  |  | conserved hypothetical protein |  | 99,00 | QIDVEVIGDR | Gln->pyro-Glu@N-term |
|  |  |  |  |  |  |  |  | 99,00 | YIQATDLSEASR |  |
|  |  |  |  |  |  |  |  | 98,00 | TTYEEIKK |  |
|  |  |  |  |  |  |  |  | 2,00 | MLYSSSFDALK | Oxidation(M)@1 |
|  |  |  |  |  |  |  |  | 99,00 | QIDVEVIGDR |  |
| **0599** | 17 | AAEL003393-PA | Q17FL3_AEDAE | 53,974 | 5,02 | atp synthase beta subunit | 9 | 99,00 | AHGGYSVFAGVGER |  |
|  | 0 | AAEL002827-PA | Q17H12_AEDAE | 53,912 | 5,03 | atp synthase beta subunit |  | 99,00 | DQEGQDVLLFIDNIFR |  |
|  |  |  |  |  |  |  |  | 99,00 | IINVIGEPIDER |  |
|  |  |  |  |  |  |  |  | 99,00 | IPVGAETLGR |  |
|  |  |  |  |  |  |  |  | 99,00 | LVLEVAQHLGENTVR |  |
|  |  |  |  |  |  |  |  | 99,00 | VALTGLTVAEYFR |  |
|  |  |  |  |  |  |  |  | 99,00 | VLDTGSPIRIPVGAETLGR |  |
|  |  |  |  |  |  |  |  | 99,00 | VVDLLAPYAK |  |
|  |  |  |  |  |  |  |  | 90,00 | VALVYGQMNEPPGAR | Oxidation(M)@8 |
| **0600** | 8.29 | AAEL000143-PA | Q17Q52_AEDAE | 31,361 | 6,91 | conserved hypothetical protein | 6 | 99,00 | ANDTQSYIATISPAR |  |
|  |  |  |  |  |  |  |  | 99,00 | AQIPIFSQR |  |
|  |  |  |  |  |  |  |  | 99,00 | ELGIYLIGGTIPER |  |
|  |  |  |  |  |  |  |  | 89,00 | EGEETVLADLDFAKVDEVR |  |
|  |  |  |  |  |  |  |  | 83,00 | SQNVANALTR |  |
|  |  |  |  |  |  |  |  | 70,00 | IGIGICYDIR | Carbamidomethyl(C)@6 |
| **0612** | 9.52 | AAEL005766-PB | Q178U8_AEDAE | 39,484 | 8,45 | fructose-bisphosphate aldolase | 5 | 99,00 | NTPSYQAILENANVLAR |  |
|  | 0 | AAEL005766-PA | Q178U9_AEDAE | 39,12 | 8,02 | fructose-bisphosphate aldolase |  | 99,00 | QLLFTADDR |  |
|  |  |  |  |  |  |  |  | 99,00 | TTYFNYPPK | Protein Terminal Acetyl@N-term |
|  |  |  |  |  |  |  |  | 99,00 | YASVCQSQR | Carbamidomethyl(C)@5 |
|  |  |  |  |  |  |  |  | 97,00 | TTYFNYPPKDVQEELAR | Protein Terminal Acetyl@N-term |
|  | 6.47 | AAEL004930-PA | Q17BL7_AEDAE | 31,307 | 5,73 | carbonic anhydrase | 4 | 99,00 | EPIEVSHDQLELFR |  |
|  |  |  |  |  |  |  |  | 99,00 | SFAEAAGQPDGLAVLGVFLK |  |
|  |  |  |  |  |  |  |  | 99,00 | VVNNYRPPLELGNR |  |
|  |  |  |  |  |  |  |  | 66,00 | VGRPHPELDTIAK |  |
| **0618** | 6 | AAEL004930-PA | Q17BL7_AEDAE | 31,307 | 5,73 | carbonic anhydrase | 3 | 99,00 | EPIEVSHDQLELFR |  |
|  |  |  |  |  |  |  |  | 99,00 | SFAEAAGQPDGLAVLGVFLK |  |
|  |  |  |  |  |  |  |  | 99,00 | VVNNYRPPLELGNR |  |
|  | 4.54 | AAEL009462-PA | Q16VR2_AEDAE | 29,367 | 5,91 | hydroxyacylglutathione hydrolase | 3 | 99,00 | FFEGTPQQMYDALITK | Oxidation(M)@9 |
|  |  |  |  |  |  |  |  | 99,00 | FGNTVEPDNVDTLQLLNIAK |  |
|  |  |  |  |  |  |  |  | 71,00 | VVFTGDTLFLAGCGR | Carbamidomethyl(C)@13 |
| **0623** | 6 | AAEL000641-PB | Q1HR78_AEDAE | 55,954 | 4,95 | protein disulfide isomerase | 3 | 99,00 | AVFDGEYTEEALKK |  |
|  | 0 | AAEL000641-PA |  |  |  | protein disulfide isomerase |  | 99,00 | FVTAQALPLIVDFSHETAQK |  |
|  |  |  |  |  |  |  |  | 99,00 | NGTPIEYTGGR | Deamidated(N)@1 |
|  | 4 | AAEL006885-PA | 1433Z_AEDAE | 28,228 | 4,78 | 14-3-3 protein sigma, gamma, zeta, beta/alpha | 2 | 99,00 | SVTETGVELSNEER |  |
|  |  |  |  |  |  |  |  | 99,00 | YLAEVATGETR |  |
|  |  |  |  |  |  |  |  |  |  |  |
| **0627** | 12 | AAEL000641-PB | Q1HR78_AEDAE | 55,954 | 4,95 | protein disulfide isomerase | 6 | 99,00 | AFLATANAVDDYPFAVTSSEDVYAK |  |
|  | 0 | AAEL000641-PA |  |  |  | protein disulfide isomerase |  | 99,00 | AVFDGEYTEEALKK |  |
|  |  |  |  |  |  |  |  | 99,00 | EHNVAVVGFFK |  |
|  |  |  |  |  |  |  |  | 99,00 | ELETVAEAEEFLK |  |
|  |  |  |  |  |  |  |  | 99,00 | FVTAQALPLIVDFSHETAQK |  |
|  |  |  |  |  |  |  |  | 99,00 | NGTPIEYTGGR | Deamidated(N)@1 |
| **0660** | 10.85 | AAEL012996-PA | Q1HQJ5_AEDAE | 23,089 | 5,08 | rho guanine dissociation factor | 7 | 99,00 | IIFDDSDPR |  |
|  |  |  |  |  |  |  |  | 99,00 | IIFDDSDPRK |  |
|  |  |  |  |  |  |  |  | 99,00 | IRIDFIVQR |  |
|  |  |  |  |  |  |  |  | 99,00 | TIEEIMAADAEDESLR | Oxidation(M)@6 |
|  |  |  |  |  |  |  |  | 99,00 | YKEALLGEAQSEK |  |
|  |  |  |  |  |  |  |  | 86,00 | TIEEIMAADAEDESLRK | Oxidation(M)@6 |
|  |  |  |  |  |  |  |  | 58,00 | TIEEIMAADAEDESLRK | Dethiomethyl(M)@6 |
| **0685** | 10 | AAEL011627-PA | Q16PI0_AEDAE | 29,194 | 8,56 | ribose-5-phosphate isomerase | 6 | 99,00 | AYFGLADGSVTER |  |
|  |  |  |  |  |  |  |  | 99,00 | DNTVVGVGSGSTVVYAVQR |  |
|  |  |  |  |  |  |  |  | 99,00 | FDWDVVNR | Oxidation(W)@3 |
|  |  |  |  |  |  |  |  | 99,00 | GIPIEVVPMAYVPIR | Dethiomethyl(M)@9 |
|  |  |  |  |  |  |  |  | 99,00 | LVCIPTSFQAR | Carbamidomethyl(C)@3 |
|  |  |  |  |  |  |  |  | 87,00 | FDWDVVNR |  |
| **0688** | 6.46 | AAEL002542-PA | Q17HW3_AEDAE | 26,322 | 5,98 | triosephosphate isomerase | 4 | 99,00 | DLNIGWVILGHSER |  |
|  |  |  |  |  |  |  |  | 99,00 | EAGQTEAVCFR | Carbamidomethyl(C)@9 |
|  |  |  |  |  |  |  |  | 99,00 | VIACIGETLQER | Carbamidomethyl(C)@4 |
|  |  |  |  |  |  |  |  | 65,00 | TATPEQAQEVHAALR |  |
| **0697** | 3.4 | AAEL010582-PA | Q16SH7_AEDAE | 25,896 | 5,69 | glutathione-s-transferase theta, gst | 2 | 99,00 | AILIYLVEK |  |
|  |  |  |  |  |  |  |  | 96,00 | VAAEIDFSKYPNIER |  |
|  |  |  |  |  |  |  |  |  |  |  |
| **0721** | 10 | AAEL011264-PA | Q16QJ8_AEDAE | 24,974 | 5,69 | phosphatidylethanolamine-binding protein | 5 | 99,00 | IAFVGSGPPQGSGLHR |  |
|  |  |  | Q1HQH6_AEDAE | 25,002 | 5,87 |  |  | 99,00 | KYNLGELVAGNFYR |  |
|  | 0 | AAEL015260-PA | Q16ED2_AEDAE | 14,116 | 8,73 | phosphatidylethanolamine-binding protein, putative |  | 99,00 | QTDGRVDLSEAPR |  |
|  |  |  |  |  |  |  |  | 99,00 | VSYPSGVFANGGDELTPTQVK |  |
|  |  |  |  |  |  |  |  | 99,00 | YNLGELVAGNFYR |  |
|  | 3.52 | AAEL013528-PA | Q8WSF6_AEDAE | 21,862 | 5,67 | peroxiredoxins, prx-1, prx-2, prx-3 | 2 | 99,00 | QITINDLPVGR |  |
|  |  |  |  |  |  |  |  | 97,00 | GLFIIDPNGVVR |  |
| **0725** | 4 | AAEL004112-PA | Q8WSF6_AEDAE | 21,862 | 5,67 | peroxiredoxins, prx-1, prx-2, prx-3 | 3 | 99,00 | DYGVLQEESGVPFR |  |
|  |  |  |  |  |  |  |  | 99,00 | QVTVNDLPVGR | Gln->pyro-Glu@N-term |
|  |  |  |  |  |  |  |  | 99,00 | QVTVNDLPVGR |  |
|  | 3.05 | AAEL011741-PC | Q16P80_AEDAE | 23272 | 5,18 | glutathione s-transferase | 2 | 99,00 | FLLSYGNLPFDDIR |  |
|  | 0 | AAEL011741-PA | Q16P78_AEDAE | 23278 | 4,98 | glutathione s-transferase |  | 91,00 | LVTLNSEVIPFYLEK |  |
|  | 0 | AAEL011741-PB | Q16P79_AEDAE | 26994 | 5,24 | glutathione s-transferase |  |  |  |  |
| **0728** | 12.54 | AAEL011741-PC | Q16P80_AEDAE | 23272 | 5,18 | glutathione s-transferase | 8 | 99,00 | FLLSYGNLPFDDIR |  |
|  | 0 | AAEL011741-PA | Q16P79_AEDAE | 26994 | 5,24 | glutathione s-transferase |  | 99,00 | IAVVSYEPDDDVK |  |
|  |  |  |  |  |  |  |  | 99,00 | IAVVSYEPDDDVKEK |  |
|  |  |  |  |  |  |  |  | 99,00 | KLVTLNSEVIPFYLEK |  |
|  |  |  |  |  |  |  |  | 99,00 | LVTLNSEVIPFYLEK |  |
|  |  |  |  |  |  |  |  | 99,00 | SDLVANHPNLQR |  |
|  |  |  |  |  |  |  |  | 71,00 | VYYFNVK | Iodo(Y)@3 |
|  |  |  |  |  |  |  |  | 99,00 | FLLSYGNLPFDDIR | Oxidation(Y)@5 |
| **0747** | 6 | AAEL013528-PA | Q16IW1_AEDAE | 25,881 | 8,68 | peroxiredoxins, prx-1, prx-2, prx-3 | 4 | 99,00 | GLFIIDPNGVVR |  |
|  |  |  |  |  |  |  |  | 99,00 | HGEVCPANWDPK | Carbamidomethyl(C)@5 |
|  |  |  |  |  |  |  |  | 99,00 | QITINDLPVGR | Gln->pyro-Glu@N-term |
|  |  |  |  |  |  |  |  | 99,00 | QITINDLPVGR |  |
| **0785** | 8.82 | AAEL008303-PA | Q16Z50_AEDAE | 19,049 | 7,76 | calponin/transgelin | 5 | 99,00 | ANEGQLNLQMGYNK | Oxidation(M)@10 |
|  |  |  |  |  |  |  |  | 99,00 | LPPGAYEDVLK |  |
|  |  |  |  |  |  |  |  | 99,00 | NIPQVTLCLYSLGR | Carbamidomethyl(C)@8 |
|  |  |  |  |  |  |  |  | 99,00 | YGVPEEEIFQTADLFER |  |
|  |  |  |  |  |  |  |  | 85,00 | TFTEEQLR |  |
| **0792** | 3.4 | AAEL011288-PA | Q16QH0_AEDAE | 48,94 | 7,51 | elongation factor 1 gamma | 2 | 99,00 | GTFNFDDFKR |  |
|  |  |  |  |  |  |  |  | 96,00 | SKDPFDSLPK |  |
| **0804** | 8 | AAEL003957-PC | Q1HQF5_AEDAE | 17,105 | 6,74 | conserved hypothetical protein | 6 | 99,00 | NAEYDQFLEDIQK |  |
|  | 0 | AAEL003957-PB |  |  |  | conserved hypothetical protein |  | 99,00 | QIDVEVIGDR | Gln->pyro-Glu@N-term |
|  | 0 | AAEL003957-PA |  |  |  | conserved hypothetical protein |  | 99,00 | YIQATDLSEASR |  |
|  |  |  |  |  |  |  |  | 99,00 | YVIFYIRDEK |  |
|  |  |  |  |  |  |  |  | 99,00 | NAEYDQFLEDIQK | Iodo(Y)@4 |
|  |  |  |  |  |  |  |  | 99,00 | QIDVEVIGDR |  |
| **0808** | 9.71 | AAEL003957-PC | Q1HQF5_AEDAE | 17,105 | 6,74 | conserved hypothetical protein | 7 | 99,00 | MLYSSSFDALKK | Oxidation(M)@1 |
|  | 0 | AAEL003957-PB |  |  |  | conserved hypothetical protein |  | 99,00 | NAEYDQFLEDIQK |  |
|  | 0 | AAEL003957-PA |  |  |  | conserved hypothetical protein |  | 99,00 | QIDVEVIGDR | Gln->pyro-Glu@N-term |
|  |  |  |  |  |  |  |  | 99,00 | YIQATDLSEASR |  |
|  |  |  |  |  |  |  |  | 98,00 | TTYEEIKK |  |
|  |  |  |  |  |  |  |  | 2,00 | MLYSSSFDALK | Oxidation(M)@1 |
|  |  |  |  |  |  |  |  | 99,00 | QIDVEVIGDR |  |
| **0815** | 10 | AAEL003957-PC | Q1HQF5_AEDAE | 17,105 | 6,74 | conserved hypothetical protein | 6 | 99,00 | MLYSSSFDALKK | Oxidation(M)@1 |
|  | 0 | AAEL003957-PB |  |  |  | conserved hypothetical protein |  | 99,00 | NAEYDQFLEDIQK |  |
|  | 0 | AAEL003957-PA |  |  |  | conserved hypothetical protein |  | 99,00 | QIDVEVIGDR | Gln->pyro-Glu@N-term |
|  |  |  |  |  |  |  |  | 99,00 | YIQATDLSEASR |  |
|  |  |  |  |  |  |  |  | 99,00 | YVIFYIRDEK |  |
|  |  |  |  |  |  |  |  | 99,00 | QIDVEVIGDR |  |
