# Supplementary material for: Differential Protein Modulation in Midguts of Aedes aegypti Infected with Chikungunya and Dengue 2 Viruses
Source: PLoS One. 2010 Oct 5;5(10):e13149. doi: 10.1371/journal.pone.0013149 (PMC2950154; doi:10.1371/journal.pone.0013149)
Supplement: Table S3 — Summary of proteins identified as being modulated after midgut infection by CHIKV or DENV-2. (0.32 MB DOC) [file pone.0013149.s003.doc]

Table S3 : Summary of proteins identified as being modulated after midgut infection by CHIKV or DENV-2

| N°ID | Spot number  modulation dengue/control | Spot number/modulation DENV2/control (DC)  CHIKV/control (CC) | Spot number/  modulation DENV-2/CHIKV | Spot number  modulationCHIKV/control | ID (Vectorbase) | Definition | Localization | Comments |
| --- | --- | --- | --- | --- | --- | --- | --- | --- |
| 0008 | 74  up regulated |  |  |  | 010382 | Aldehyde oxidase | ubiquitous | *Reactive oxygen species-generating enzyme*  *Iron ion binding* |
| 0012 | 59  up regulated |  |  |  | 010382 | Aldehyde oxidase  avec modif |  |  |
| 0013 | 51  up regulated |  |  |  | 010382 | Aldehyde oxidase |  |  |
| 0014 | 54  up regulated |  |  |  | 010382 | Aldehyde oxidase  avec modif |  |  |
| 0015 | 61  up regulated |  |  |  | 010382 | Aldehyde oxidase  avec modif |  |  |
| ID0017 | 32  up regulated | 38  up regulated DC |  |  | 010382 | Aldehyde oxidase |  |  |
| 0018 | 6  up regulated | 11  up regulated DC | 12  up regulated |  | 009691 | Carboxylase:pyruvate/acetylcoa/propionyl-coa | cytoplasmic | *Catalyses the ATP-dependent carboxylation of pyruvate to oxaloacetate, thus playing a crucial role in gluconeogenesis* |
| 0020 |  |  |  | 42  up regulated | 010382 | Aldehyde oxase |  |  |
| 0021 | 80  up regulated |  |  |  | 009185  006693-PA, PB | Arginine or creatine kinase  Uroporphyrinogen decarboxylase | cytoplasmic  ? | *Protein modification*  *Heme biosynthesis* |
| 0023 | 4  up regulated | 8  up regulated DC | 9  up regulated |  | 009185 | Arginine or creatine kinase | cytoplasmic |  |
| 0024 |  |  |  | 27  up regulated | NI | - |  |  |
| 0027 | 3  up regulated |  | 3  up D/C |  | NI | - |  |  |
| 0033 | 2  up regulated |  | 7  up regulated |  | 009185 | Arginine or creatine kinase | cytoplasmic | *Protein modification* |
| 0034 | 9  up regulated | 16  up regulated DC | 15  up regulated |  | NI | - |  |  |
| 0035 | 1  up regulated | 1  up regulated DC  down regulated CC | 1  up regulated |  | NI | - |  |  |
| 0075 | 83  down regulated | 139  down regulated DC |  |  | 012278  007254 | Metalloprotease  Metalloprotease | ? | *Protein modification* |
| 0076 | 98  down regulated | 144  down regulated DC |  |  | 012278 | Metalloprotease | ? | « |
| 0077 | 158  up regulated | 173  up regulated DC and CC |  | 48  up regulated | NI | - |  |  |
| 0079 |  | 47  down regulated DC |  |  | NI | - |  |  |
| 0081 | 100  up regulated | 147  up regulated DC | 112  up regulated |  | NI | - |  |  |
| 0087 | 86  down regulated | 56  down regulated DC |  |  | 007201 | Glutamyl aminopeptidase | secreted | *Zn dependent membrane bound aminopeptidase that catalyzes the cleavage of glutamic and aspartic amino acids*  *Gene differentially expressed in Ae.aegypti during heat shock treatment (response to stress)*  *Expression increased in virus infected pre-B cells*  *Protein modification* |
| 0093 | 18  up regulated | 34  up regulated DC |  |  | 005429 | 2-oxoglutarate dehydrogenase |  | *Tricarboxylic acid cycle*  *Differentially expressed in blood fed Triatoma* |
| 0094 | 201  up regulated | 169  up regulated DC  up regulated CC |  | 68  up regulated | 008216 | Aconitase | ? | *Iron sulfur protein with non-redox functions*  *Involved in the tricarboxylic ac cycle*  *May play a role in regulating resistance to oxative stress*  *Participates to ribosome binding and mRNA turnover*  *Energy production* |
| 0095 | 64  up regulated | 107  up regulated DC  up regulated CC |  | 48  up regulated | 008216 | Aconitase modif |  | « |
| 0096 | 22  up regulated | 39  up regulated DC  up regulated CC |  |  | 005429 | 2-oxoglutarate dehydrogenase modif |  | *Tricarboxylic cycle*  *Energy production* |
| 0108 | 30  down regulated |  |  |  | 012918 | Puromycin-sensitive aminopeptidase | ? | *Participate in variety of proteolytic events essential for cell growth and viability, and in fertility in abroad range of organisms*  *Protein metabolism* |
| 0112 | 20  down regulated |  |  |  | 007201 | Glutamyl aminopeptidase |  | *Amino acid metabolism* |
| 0146 | 179  up regulated | 178  up regulated DC  up regulated CC |  |  | 002870-PA and PB | Dipeptyl peptidase III (DPP III) | cytoplasmic | *Proteolysis*  *hydrolyses small peptes with a broad substrate specificity.*  *Protein metabolism*  *It is thought to be involved in a major degradation*  *pathway of the insect neuropepte proctolin*  *which is implied in hindgut contraction*  *Protein modification* |
| 0154 | 14  up regulated | 30  up regulated DC  up regulated CC |  |  | NI | - |  |  |
| 0156 | 24  up regulated | 44  up regulated DC  up regulated CC |  |  | NI | - |  |  |
| 0160 |  |  | 81  up regulated |  | NI | - |  |  |
| 0163 |  |  | 111  down regulated |  | 008216  007915 | Aconitase  Moesin/ezrin/radixin | cytoplasmic | *Iron sulfur protein with non-redox functions*  *Involved in the tricarboxylic ac cycle*  *Cytoskeletal protein binding*  *Microtubule organization* |
| 0188 |  |  | 13  up regulated |  | NI | - |  |  |
| 0189 |  |  | 24  up regulated |  | NI | - |  |  |
| 0191 | 121  down regulated | 142  down regulated DC | 96  down regulated |  | NI | - |  |  |
| 0192 |  | 37  up regulated DC  down regulated CC | 28  up regulated | 22  down regulated | NI | - |  |  |
| 0195 | 34  up regulated | 36  up regulated DC | 27  up regulated |  | 004369 | Alpha-glucosidase | secreted | [*glucosase*](http://en.wikipedia.org/wiki/Glucosidase) *acting upon 1,4-alpha bonds*  *inhibitors eliminate the production of several endoplasmic reticulum-budding viruses like D2*  *Carbohydrate metabolism* |
| 0196 | 72  up regulated | 120  up regulated DC |  |  | NI | - |  |  |
| 0199 | 85  down regulated | 141  down regulated DC down regulated CC |  |  | 015458  015639 | Transferrin  Transferrin | secreted | *Implied in* [*iron*](http://en.wikipedia.org/wiki/Iron) [*ion*](http://en.wikipedia.org/wiki/Ion) *delivery*  *Several reports have described up-regulation of transferrins in insects or insect cells challenged with bacteria*  *Iron transport*  *Iron transport* |
| 0201 |  | 149  up regulated CC | 103  down regulated | 73  up regulated | 004580 | Beta-galactosidase | secreted | *hydrolyses an osidic link involving a galactose in position β*  *Carbohydrate metabolism* |
| 0234 |  |  | 6  up regulated |  | 004434 | Transketolase modif |  | *Enzyme of the* [*pentose phosphate pathway*](http://en.wikipedia.org/wiki/Pentose_phosphate_pathway)  *galactose binding lectin*  *proves a link between the glycolytic and pentose-phosphate pathways*  *Increased in infected Drosophila*  *Energy production* |
| 0236 |  |  | 2  up regulated |  | 004434 | Transketolase |  | *Pentose phosphate pathway*  *Energy production* |
| 0243 |  |  |  | 90  up regulated | 011778 | Wd-repeat protein |  | *May promote maintenance of the viral genome in cells* |
| 0252 | 75  up regulated | 123  up regulated DC |  |  | 011584 | Chaperonin 60 kDa | cytosol | *ATP binding, unfolded protein binding, protein binding*  *stress-induced, acting to stabilise or protect disassembled polypeptides under heat-shock conditions*  Chaperonin 60 kDa modif  *Down regulated in sindbis virus infection*  *Protein metabolism and stress response* |
| 0253 |  |  |  | 8  up regulated | NI | - |  |  |
| 0254 | 70  up regulated | 118  up regulated DC |  |  | 011584 | Chaperonin 60 kDa modif | cytosol |  |
| 0259 | 153  up regulated |  |  |  | 011584 | Chaperonin 60 kDa modif | cytosol |  |
| 0267 | 172  up regulated |  |  |  | 005790 | Malic enzyme | mitochondria | *Lipogenesis*  *Involved in antioxidant defence*  *Involved in the tricarboxylic cycle* |
| 0268 | 68  up regulated |  |  |  | NI | - |  |  |
| 0271 | 13  up regulated | 28  up regulated DC  up regulated CC |  |  | NI | - |  |  |
| 0273 | 79  up regulated | 43  up regulated DC  down regulated CC | 35  up regulated |  | NI | - |  |  |
| 0282 | 146  up regulated | 65  up regulated DC  down regulated CC |  |  | NI | - |  |  |
| 0283 | 200  up regulated |  |  |  | 002886 PA and PB | Thioredoxin reductase |  | *Cell redox homeostasis*  *Antioxidant related* |
| 0284 |  |  | 43  up regulated |  | NI | - |  |  |
| 0290 | 177  up regulated |  |  |  | 013407 | Catalase | ? | *Response to oxidative stress*  *Antioxidant related* |
| 0291 | 120  up regulated | 160  up regulated DC |  |  | NI | - |  |  |
| 0302 |  |  | 18  up regulated |  | 000641 PA PB | Protein disulfe isomerase | secreted | *Regulates the activity of target proteins through changes in the redox state of thiol groups*  *Antioxidant related* |
| 0304 | 55  up regulated | 62  up regulated DC | 49  up regulated |  | 006823-PA | AMP dependent ligase | ? | *Metabolic process* |
| 0305 | 53  up regulated | 40  up regulated DC | 31  up regulated |  | 006823-PA | AMP dependent ligase | « | « |
| 0319 |  | 140  down regulated CC  up regulated DC | 95  up regulated | 79  down regulated | 011309  011137 | Orotidine 5’ phophate decarboxylase  Succinyl-coa :3ketoacid coenzyme A transferase | secreted | *Pyrimidine metabolism*  *involved in nucleotide synthesis and salvage*    *Important intermediate in the tricarboxylic acid cycle* |
| 0328 | 134  down regulated | 129  down regulated DC down regulated CC |  |  | NI | - |  |  |
| 0345 |  | 180  up regulated CC |  | 81  up regulated | 009387-PA and PB | Hexokinase |  | *Glycolysis*  *Energy production* |
| 0358 | 12  up regulated | 27  up regulated DC |  |  | 003746 | 4 hydroxybutyrate CoA transferase putative | ? | *Acetyl-CoA metabolic process, a key intermediate in lipid and terpeno biosynthesis*  *Lipid metabolism* |
| 0360 | 7  up regulated | 20  up regulated DC |  |  | 003746 | 4 hydroxybutyrate CoA transferase putative | ? | « |
| 0364 | 210  up regulated |  |  |  | 001668 | Enolase |  | *Glycolysis*  *Energy production* |
| 0375 |  | 151  up regulated DC  up regulated CC |  | 55  up regulated | 009872 | Alanine aminotransferase | cytoplasmic | *Amino acid metabolism* |
| 0424 |  |  |  | 64  up regulated | 012579  002160 | Aspartate amino transferase  GTP binding protein | cytoplasmic | *Amino acid metabolism*  *G proteins are important* [*signal transducing*](http://en.wikipedia.org/wiki/Signal_transducing) *molecules in cells* |
| 0433 |  |  | 30  up regulated |  | 000641-PA and PB  011197-PA, PB | Protein disulfide isomerase  Actin | endoplasmic reticulum lumen  cytoplasmic | *Protein modification*  *Defense/immunity related*  *Cytoskeleton* |
| 0442 | 15  up regulated |  |  |  | 001887-PA and PB | Glutamine synthetase 1, 2 | cytoplasmic | *Amino acid metabolism* |
| 0443 | 28  up regulated | 41  up regulated DC |  |  | 001887-PA and PB | Glutamine synthetase 1, 2 | cytoplasmic | *Amino acid metabolism* |
| 0445 | 67  up regulated | 67  up regulated DC | 56  up regulated |  | 001887-PA and PB | Glutamine synthetase 1, 2 | cytoplasmic | *Amino acid metabolism* |
| 0447 |  |  | 115  down regulated |  | 005766-PA and PB | Fructose bis phosphate aldolase | ? | *Glycolysis*  *Energy production* |
| 0467 | 108  up regulated | 153  up regulated DC |  |  | 007555 | Acyl coa dehydrogenase |  | *Lipid metabolism* |
| 0469 |  |  |  | 89  down regulated | 009185  006693-PA, PB, PC | Arginine or creatine kinase  Uroporphirinogen decarboxylase |  | *Involved in protein phosphorylation*  *Protein metabolism*  *catalyzes the fifth step in* [*heme*](http://en.wikipedia.org/wiki/Heme) *biosynthesis* |
| 0470 | 17  up regulated | 33  up regulated DC  up regulated CC |  |  | 007555 | Acyl coa dehydrogenase |  | *Catalyzes the initial step in each cycle of fatty acid β -oxation in the mitochondria of cells*  *Lipid metabolism?* |
| 0476 |  |  |  | 78  down regulated | NI | - |  |  |
| 0477 |  |  |  | 91  up regulated | 015034 | Alcohol dehydrogenase | ? | *Oxydoreduction*  *facilitate the conversion of toxic alcohols to aldehydes* |
| 0501 |  |  |  | 93  up regulated | 008006  004086-PA and PB | 3-hydroxyacyl-coA dehydrogenase  aldo keto reductase | mitochondrial  ? | [*Oxydoreductase*](http://en.wikipedia.org/wiki/Oxidoreductase) *involved in fatty acid metabolic processes*  *Part of the family of* [*NADPH*](http://en.wikipedia.org/wiki/Nicotinamide_adenine_dinucleotide_phosphate)*-dependent* [*oxydoreductases*](http://en.wikipedia.org/wiki/Oxidoreductase)  *catalyze redox transformations involved in biosynthesis, intermediary metabolism, and detoxification*  *protect cells from endogenously formed reactive carbonyl groups* |
| 0546 |  |  |  | 96  up regulated | NI | - |  |  |
| 0550 | 60  down regulated |  |  |  | NI | - |  |  |
| 0554 |  | 146  up regulated DC |  | 69  up regulated | 011302-PE, PD, PC, PB | Annexin | ? | *Calcium-dependent phospholipid binding*  *Involved in exocytic and endocytic pathways and in resistance to reactive oxygen species* |
| 0555 |  | 83  up regulated DC  up regulated CC |  |  | NI | - |  |  |
| 0568 |  |  |  | 59  down regulated | 013739  000219 | Electron transport oxoreductase  Lactoyl glutathione lyase |  | Involved in the oxydation of fatty acids  *Generates energy required for the production of ATP*  *Energy production*  *Part of the glyoxalase system*  *carries out the* [*detoxification*](http://en.wikipedia.org/wiki/Detoxification) *of* [*methylglyoxal*](http://en.wikipedia.org/wiki/Methylglyoxal) *and the other reactive* [*aldehydes*](http://en.wikipedia.org/wiki/Aldehyde)  *involved in detoxification, cell growth and microtubule assembly* |
| 0569 | 106  down regulated |  |  |  | 004930  000219 | Carbonic anhydrase  Lactoyl glutathione lyase |  | *pH regulation*  *When inhibited, involved in viral enhancement*  *Part of the glyoxalase system, implied in detoxification* |
| 0571 | 58  down regulated | 60  down regulated DC  up regulated CC |  |  | 003957-PA, PB, PC | Conserved hypothetical protein | intracellular | *Actin depolymerizing factor/cofilin, actin-binding proteins that regulate actin filament dynamics* |
| 0586 |  | 152  up regulated CC |  | 61  up regulated | NI | - |  |  |
| 0599 |  |  | 51  down regulated |  | 003393  002827 | ATP synthase beta subunit  ATP synthase beta subunit | membrane bound | *Ion transporter implied in ATP synthesis*  *Energy production* |
| 0600 | 127  down regulated | 161  down regulated DC down regulated CC |  |  | 000143 | Conserved hypothetical protein | ? | *Ortholog of carbon-nitrogen hydrolase in Ixodes*  *nitrogen compound metabolic process* |
| 0602 | 69  down regulated |  |  |  | NI | - |  |  |
| 0612 | 81  down regulated | 137  down regulated DC |  |  | 005766-PA and PB  004930 | Fructose-bisphosphate aldolase  Carbonic anhydrase |  | *Glycolysis*  *Up regulated in dengue fever patients*  *May be involved in direct interaction with RNA viruses*  *Energy production*  *pH regulation*  *When inhibited, involved in viral enhancement* |
| 0618 |  |  | 71  down regulated |  | 004930  009462 | Carbonic anhydrase  Hydroxyacylglutathione hydrolase |  | *pH regulation*  *When inhibited, involved in viral enhancement*  *Participates in pyruvate metabolism*  *Involved in the synthesis of glutathion*  *antioxidant related* |
| 0623 |  | 113  up regulated CC  down regulated DC | 86  down regulated | 87  up regulated | 000641-PA  1-PB  006885 | Protein disulfide isomerase  14.4.3 | ?  cytoplasm | *Possesses a molecular mimicry with NS1*  *Involved in ER-stress response*  *mainly binds proteins containing phosphothreonine or phosphoserine motifs*  *appears to effect intracellular signalling*  *Binds to molecular chaperone Hsp60* |
| 0627 | 84  down regulated | 72  down regulated DC  up regulated CC |  |  | 000641-PA  1-PB | Protein disulfide isomerase | endoplasmic reticulum (ER) | *Involved in ER-stress response* |
| 0660 | 214  down regulated |  |  |  | 012996 | rho guanine dissociation factor | cytoplasmic | *regulates GDP/GTP exchange*  *involved in nucleotide exchange as source of energy*  *Energy production* |
| 0666 |  |  | 26  up regulated |  | NI | - |  |  |
| 0685 |  |  | 109  down regulated |  | 011627 | Ribose-5-phosphate isomerase | secreted | *Glycolysis*  *Energy production* |
| 0688 |  | 94  down regulated DC  up regulated CC | 70  down regulated |  | 002542 | Triosephosphate isomerase | ? | *Glycolysis*  *Induced in virus infected plants*  *Energy production* |
| 0689 | 114  down regulated |  |  |  | NI | - |  |  |
| 0691 | 112  down regulated |  |  |  | NI | *-* |  |  |
| 0697 |  |  | 87  down regulated |  | 010582 | Glutathione-s-transferase theta gst | cytoplasmic and nuclear | *Transferase*  *key role in cellular detoxification*  *Implied in the defense of Anopheles mosquito to parasite infection*  *Antioxidant related* |
| 0721 |  |  | 117  down regulated |  | 011264  015260  013528 | Phosphatidylethanolamine binding protein  Phosphatidylethanolamine binding protein, putative  Peroxiredoxins, prx1, prx2 prx3 (*AsPrx-4783* | secreted  ? | *Lip binding, serine protease*    *The crystal structure of human phosphatylethanolamine-binding protein suggests a role in membrane signal transduction pathways such as the MAP kinase pathway, and the NF-kappaB pathway*  *Putative serine protease inhibitor*  *Antioxidant related*  *expression is induced in the mosquito midgut by parasite infection at times associated with peak nitrosative and oxative stresses*) |
| 0725 | 190  down regulated | 93  down regulated DC  up regulated CC |  |  | 004112  011741-PA, PB and PC | Peroxiredoxins, prx1, prx2 prx3  Glutathione-S-transferase | ?  cytosol | *Antioxidant related*  *Antioxidant related* |
| 0728 |  |  | 92  down regulated |  | 011741-PC and PA | Gluthatione-S-transferase | cytosol | *Participates in the detoxification of reactive electrophillic compounds detoxifies endogenous compounds* |
| 0747 | 82  down regulated |  |  |  | 013528 | Peroxiredoxin 1, 2, 3 |  |  |
| 0785 | 71  down regulated |  |  |  | 008303 | Calponin/transgelin | cytosol | *Actin binding protein*  *Down regulated in bacteria infected heads of Honney bee* |
| 0792 |  | 17  up regulated CC |  | 1  up regulated | 011288 | Elongation factor 1 gamma | cytosol | *Responsible for achieving accuracy of translation*  *contains GST domains* |
| 0804 |  |  | 78  down regulated | 80  up regulated | 003957 PA, PB and PC | Conserved hypothetical protein |  | *Actin-binding, cofilin/tropomyosin type* |
| 0808 | 107  down regulated |  | 20  down regulated |  | 003957 PA, PB and PC | Conserved hypothetical protein |  | *Actin-binding, cofilin/tropomyosin type* |
| 0815 |  |  | 62  down regulated | 52  up regulated | 003957 PA, PB and PC | Conserved hypothetical protein |  | *Actin-binding, cofilin/tropomyosin type* |

^a^: the fold modulation is followed by the spot number to which it is associated. NI means that no identifiation was obtained from the Vectorbase database.
