# Supplementary material for: Differential Protein Modulation in Midguts of Aedes aegypti Infected with Chikungunya and Dengue 2 Viruses
Source: PLoS One. 2010 Oct 5;5(10):e13149. doi: 10.1371/journal.pone.0013149 (PMC2950154; doi:10.1371/journal.pone.0013149)
Supplement: Table S4 — Differential expression of midgut proteins according to their role after infection by CHIKV or DENV-2 viruses. (0.15 MB DOC) [file pone.0013149.s004.doc]

Table S4: Differential expression of midgut proteins according to their role after infection by CHIKV or DENV-2 viruses.

| **Identification** | **CHIKV infection**  **Fold modulation***  **(Anova)*** | **DENV-2 infection**  **Fold modulation***  **(Anova)*** |
| --- | --- | --- |
| ***Reactive oxygen species-generating enzyme*** |  |  |
| Aldehyde oxidase | 1.8 (0.016) | 2.08 ± 0.19 (0.007± 0.006) |
| ***Antioxidant related*** |  |  |
| Malic enzyme |  | 1.4 (0.032) |
| Thioredoxin reductase |  | 1.3 (0.004) |
| Catalase |  | 1.4 (0.007) |
| Hydroxyacylglutathione hydrolase |  | -2 (0.019) / CHIKV |
| Glutathione-s-transferase theta gst |  | -1.9 (0.01) /CHIKV |
| Glutathion s transferase | 1.5 (0.016) | -1.3 (0.016) |
| Peroxiredoxins, prx1, prx2 prx3 | 1.5 (0.016) | -1.8 (0.011) |
| ***Iron transport*** |  |  |
| Transferrin | -1.7 (0.037) | -1.7 (0.008) |
| ***Detoxification*** |  |  |
| Aldo keto reductase | 1.3 (0.023) |  |
| Alcohol dehydrogenase | 1.3 (0.012) |  |
| Lactoyl glutathione lyase | -1.6 (0.007) | -1.6 (0.007) |
| ***pH regulation*** |  |  |
| Carbonic anhydrase |  | -1.6 (0.007) |
| ***Anabolic pathway*** |  |  |
| Carboxylase:pyruvate/acetylcoa/propionyl-coa |  | 4 (0.004) |
| Alanine aminotransferase | 1.6 (0.002) | 1.6 (0.002) |
| ***Metabolic processes*** |  |  |
| AMP dependent ligase |  | 2 (0.004) |
| Conserved hypothetical protein 000143 | -1.6 (0.008) | -1.6 (0.001) |
| ***Nucleotide metabolism*** |  |  |
| Orotidine 5’ phophate decarboxylase | -1.4 (0.001) | 1.6 (0.002) |

Table S4: Differential expression of midgut proteins according to their role after infection by CHIKV or DENV-2 viruses (continued).

| **Identification** | **CHIKV infection**  **Fold modulation***  **(Anova)*** | **DENV-2 infection**  **Fold modulation***  **(Anova)*** |
| --- | --- | --- |
| ***Protein metabolism- modification*** |  |  |
| Arginine or creatine kinase | -1.3 (0.007) | 1.8 (0.004) |
| Puromycin-sensitive aminopeptidase |  | -2.5 (0.002) |
| Metalloprotease 012278 |  | -1.7 (5.4x10^-5^) |
| Glutamyl aminopeptidase |  | -1.7 (4x10^-4^) |
| Metalloprotease 007254 |  | -1.8 (5x10^-5^) |
| Dipeptidyl peptidase III | 1.4 (0.035) | 1.4 (0.013) |
| Aspartate amino transferase | 1.5 (0.013) |  |
| ***Protein metabolism, modification - stress response*** |  |  |
| Chaperonin 60 kDa |  | 1.7±0.16 (0.016±0.01) |
| 14-3-3 protein sigma, gamma, zeta, beta/alpha | 1.3 (0.006) | -1.9 (0.001) |
| Protein disulfide isomerase | 2.2 (0.02) | -1.7±0.36 (0.018±0,02) |
| ***Amino acid metabolism*** |  |  |
| Alanine aminotransferase | 1.6 (0.002) | 1.6 (0.002) |
| Glutamine synthetase |  | 3 (0.033) |
| Aspartate amino transferase | 1.5 (0.013) |  |
| ***Carbohydrate metabolism*** |  |  |
| Alpha-glucosidase |  | 2.63 (0.023) |
| Beta-galactosidase | 1.6±0.14 (1.5x10^-3^±0,001) |  |
| ***Lipid metabolism*** |  |  |
| Acyl coa dehydrogenase | 2.8 (0.003) | 2.2±0.6 (0.01±0.01) |
| 4 hydroxybutyrate CoA transferase putative |  | 3.4±0.2 (0.004±0.003) |
| 3-hydroxyacyl-coA dehydrogenase | 1.3 (0.023) |  |
| ***Heme biosynthesis*** |  |  |
| Uroporphirinogen decarboxylase | -1.3 (0.007) | 1,8 (0.004) |
| ***Pyrimidine metabolism*** |  |  |
| Orotidine 5’ phophate decarboxylase | -1.7±0,18 (0.001±0.001) | 1.8 (2.17 x10^-4^) |

Table S4: Differential expression of midgut proteins according to their role after infection by CHIKV or DENV-2 viruses (continued).

| **Identification** | **CHIKV infection**  **Fold modulation***  **(Anova)*** | **DENV-2 infection**  **Fold modulation***  **(Anova)*** |
| --- | --- | --- |
| ***Energy production machinery*** |  |  |
| Tricarboxylic cycle |  |  |
| Succinyl-coa :3ketoacid coenzyme A transferase | -1.7 (0.001) | 1.8 (2.5x10^-4^) |
| Aconitase | 1.65±0.15 (0.006±0.005) | 1.6±0,25 (0.002±0.001) |
| 2-oxoglutarate dehydrogenase | 2.7 (0.005) | 2.7 (0.003±0.002) |
| Glycolysis |  |  |
| Enolase |  | 1.3 (0.013) |
| Fructose-bisphosphate aldolase |  | -1.8 (3.9x10^-3^±0.003) |
| Hexokinase | 1.4 (0.004) |  |
| Triosephosphate isomerase | 1.3 (0.013) | -2 (0.008±0.005) |
| Ribose-5-phosphate isomerase |  | -1.5 (0.016) /CHIKV |
| Pentose phosphate pathway |  |  |
| Transketolase |  | 7.8±2.1 (0.006±0.005) /CHIKV |
| Transporter |  |  |
| Electron transport oxidoreductase | -1.6 (0.007) |  |
| ATP synthase beta subunit |  | -2.3 (0.018) /CHIKV |
| Nucleotide exchange |  |  |
| rho guanine dissociation factor |  | -1.3 (0.048) |
| ***Translation machinery*** |  |  |
| Elongation factor 1 gamma | 1.6 (0.007) |  |
| ***Cytoskeleton and cytoplasmic transport*** |  |  |
| Moesin/ezrin/radixin |  | -1.4 (0.016) /CHIKV |
| Actin |  | 2.7 (0.012) /CHIKV |
| Calponin/transgelin |  | -1.9 (0,011) |
| Conserved hypothetical protein 003957 | 1,55±0.015 (0.023±0.003) | -1.6 (0.016) |
| ***Signal transduction*** |  |  |
| GTP binding protein | 1.5 (0.013) |  |
| Phosphatidylethanolamine binding protein |  | -1.3 (0.017) /CHIKV |

Table S4: Differential expression of midgut proteins according to their role after infection by CHIKV or DENV-2 viruses (continued).

| **Identification** | **CHIKV infection**  **Fold modulation***  **(Anova)*** | **DENV-2 infection**  **Fold modulation***  **(Anova)*** |
| --- | --- | --- |
| ***Cytoskeleton and cytoplasmic transport*** |  |  |
| Moesin/ezrin/radixin |  | -1.4 (0.016) /CHIKV |
| Actin |  | 2.7 (0.012) /CHIKV |
| Calponin/transgelin |  | -1.9 (0,011) |
| Conserved hypothetical protein 003957 | 1.55±0.015 (0.023±0.003) | -1.6 (0.016) |
| ***Signal transduction*** |  |  |
| GTP binding protein | 1.5 (0.013) |  |
| Phosphatidylethanolamine binding protein |  | -1.3 (0.017) /CHIKV |
| ***Endocytosis exocytosis*** |  |  |
| Annexin | 1.5 (0.024) | 1.7 (0.24) |
| ***Unknown function*** |  |  |
| Wd-repeat protein | 1.4 (0.047) |  |

Proteins are grouped according to their putative function. The up and down modulations were assessed from control/CHIKV, control DENV-2 and control/CHIKV/DENV-2 gel comparisons. The type of modulation observed after CHIKV/DENV-2 gel comparisons are specified.

* mean and standard deviation values are indicated when the protein was found in several spots or when its modulation was observed in various comparisons (control/CHIKV, control/DENV-2, control/CHIKV/DENV-2). The sign “–“ was added before the fold modulation in case of down-regulation compared to the control.
