# Supplementary figures and images for: Differential Protein Modulation in Midguts of Aedes aegypti Infected with Chikungunya and Dengue 2 Viruses
Source: PLoS One. 2010 Oct 5;5(10):e13149. doi: 10.1371/journal.pone.0013149 (PMC2950154; doi:10.1371/journal.pone.0013149)

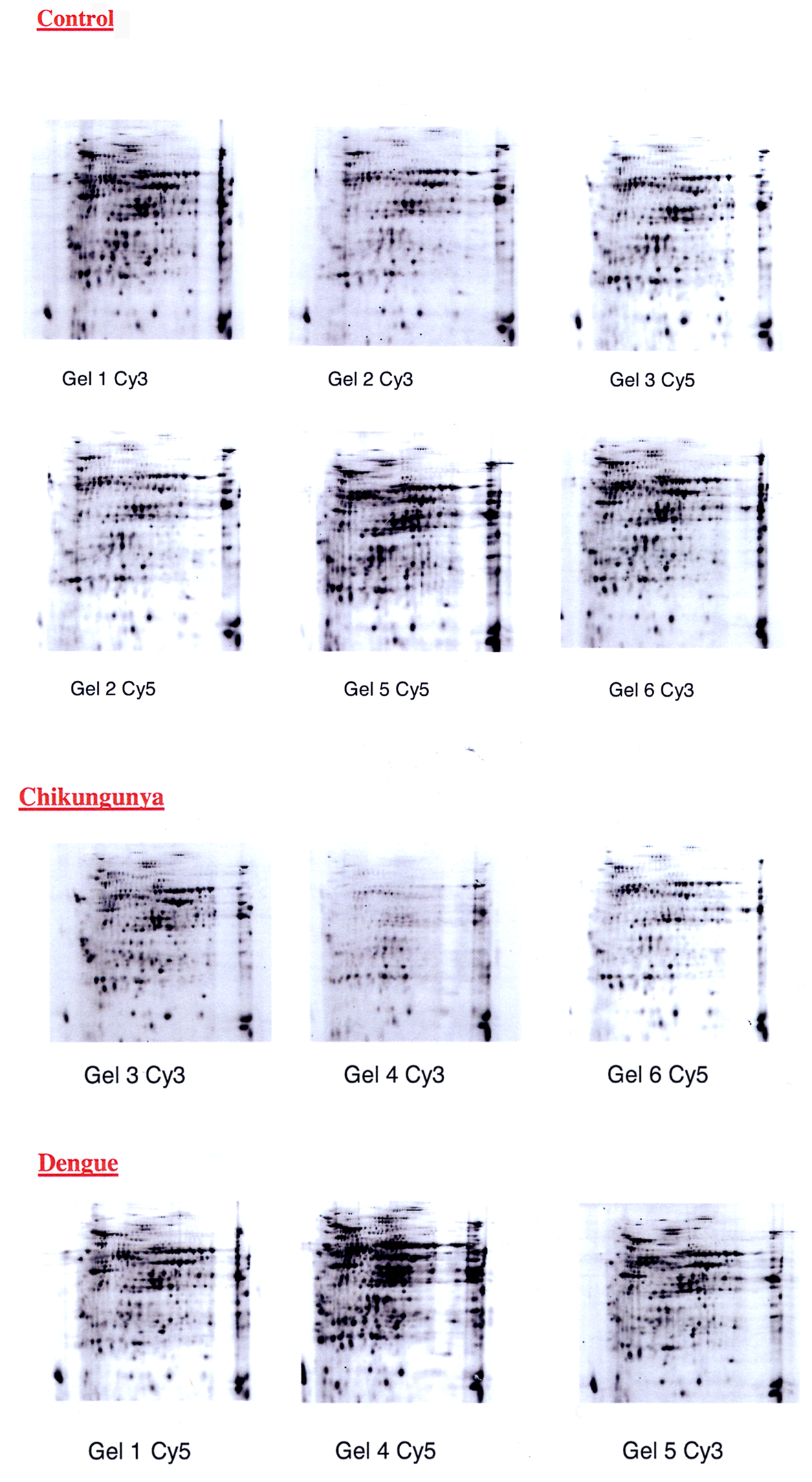

Supplement: Figure S1 — 2D-DIGE gels run with control, CHIKV- and DENV-2- infected Ae. aegypti midgut extracts. Pools of 50 µg of control blood-fed, CHIKV blood-fed and DENV-2 blood-fed midgut extracts were processed by 2D-DIGE. The gels were performed according to the protocole described in Table S1. (7.66 MB TIF) [file pone.0013149.s005.tif]

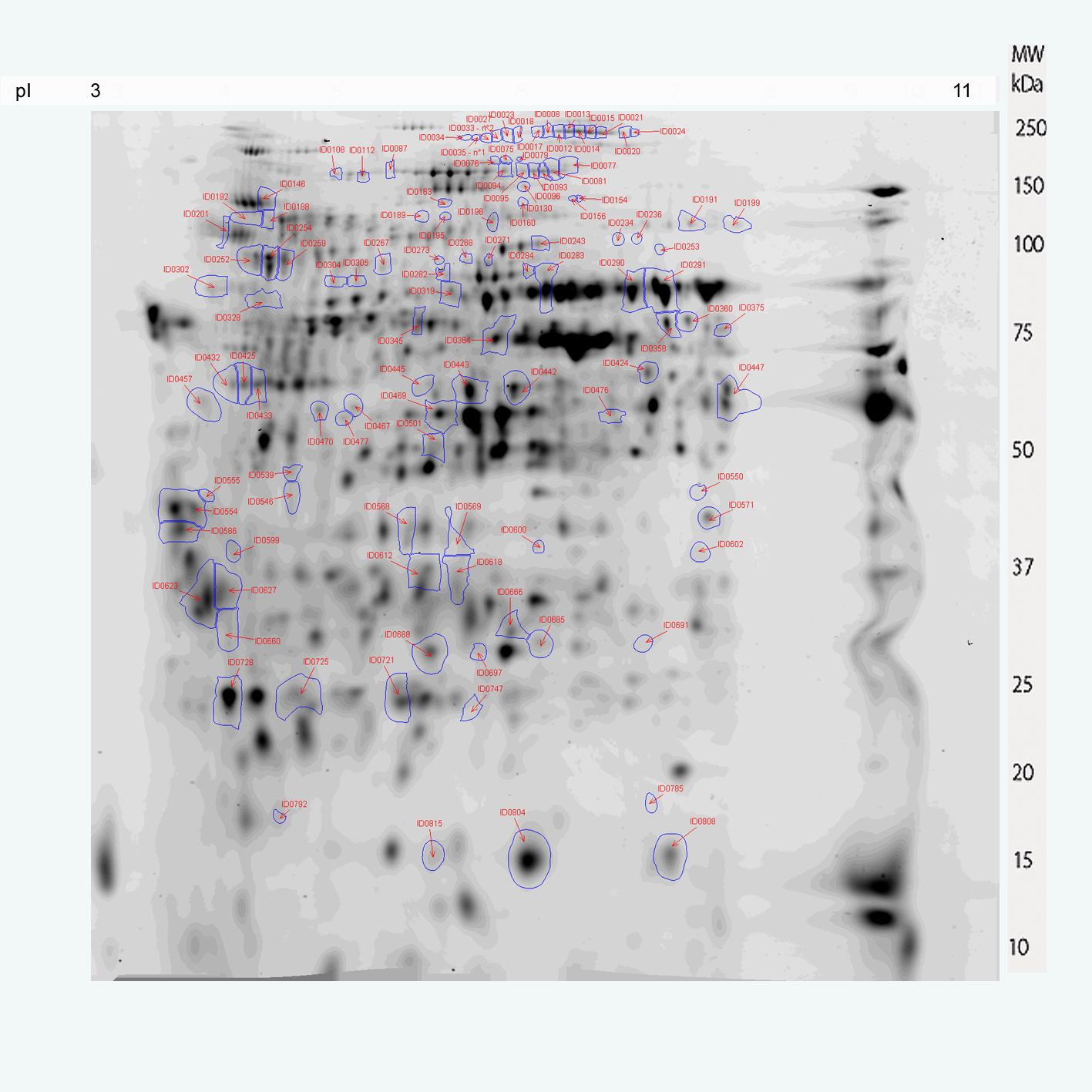

Supplement: Figure S2 — 2D-DIGE synthetic gel of Ae. Aegypti midgut extracts. Protein spots differentially expressed by both viruses are indicated by number. (6.04 MB TIF) [file pone.0013149.s006.tif]

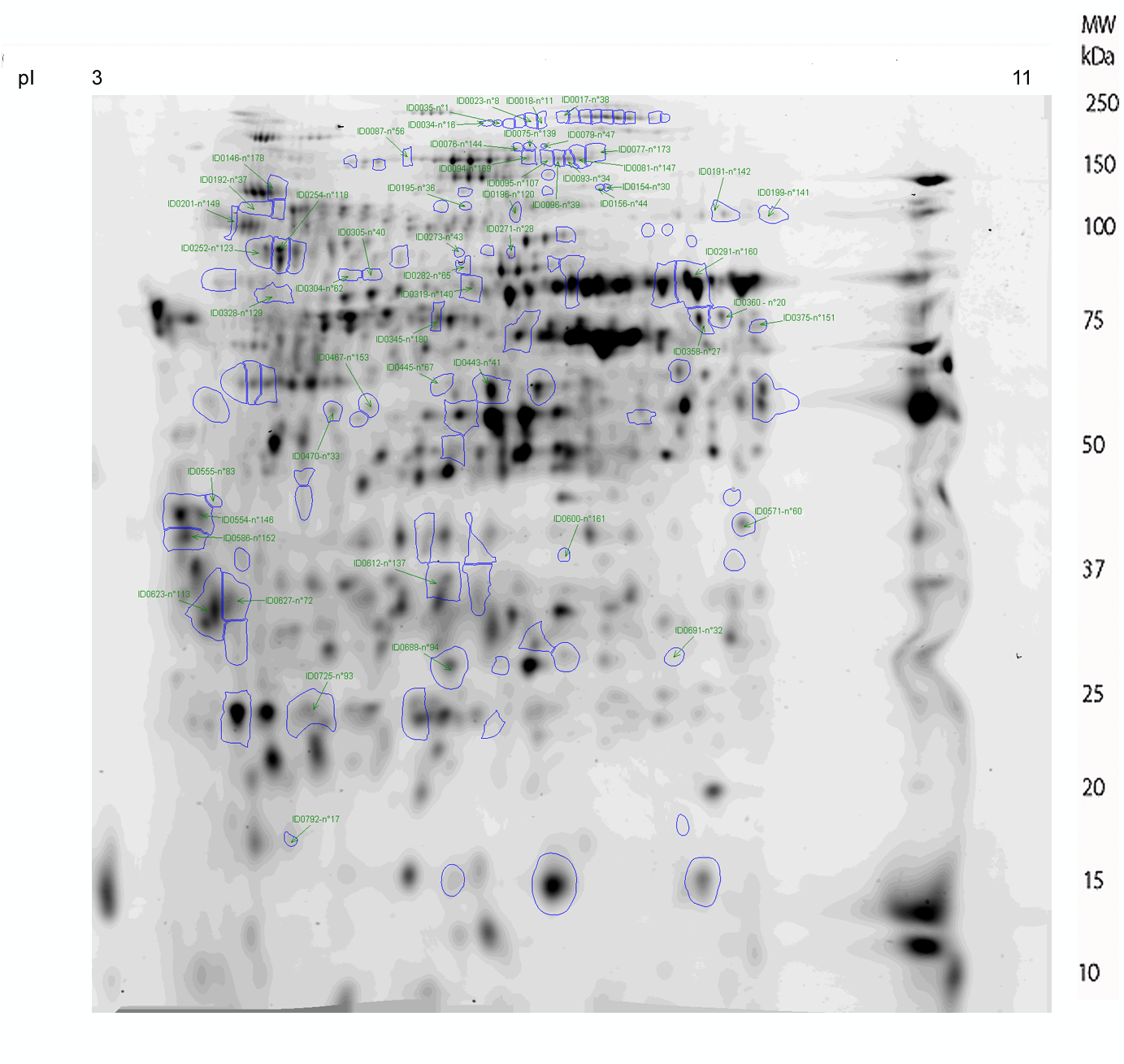

Supplement: Figure S3 — 2D-DIGE synthetic gel of Ae. aegypti midgut extracts showing spots modulated after analysis of control/CHIKV/DENV-2 profiles. Identification numbers (ID) and the range of each spot is shown on the gel. The pI and molecular weight scales are indicated in the Figure. (5.45 MB TIF) [file pone.0013149.s007.tif]

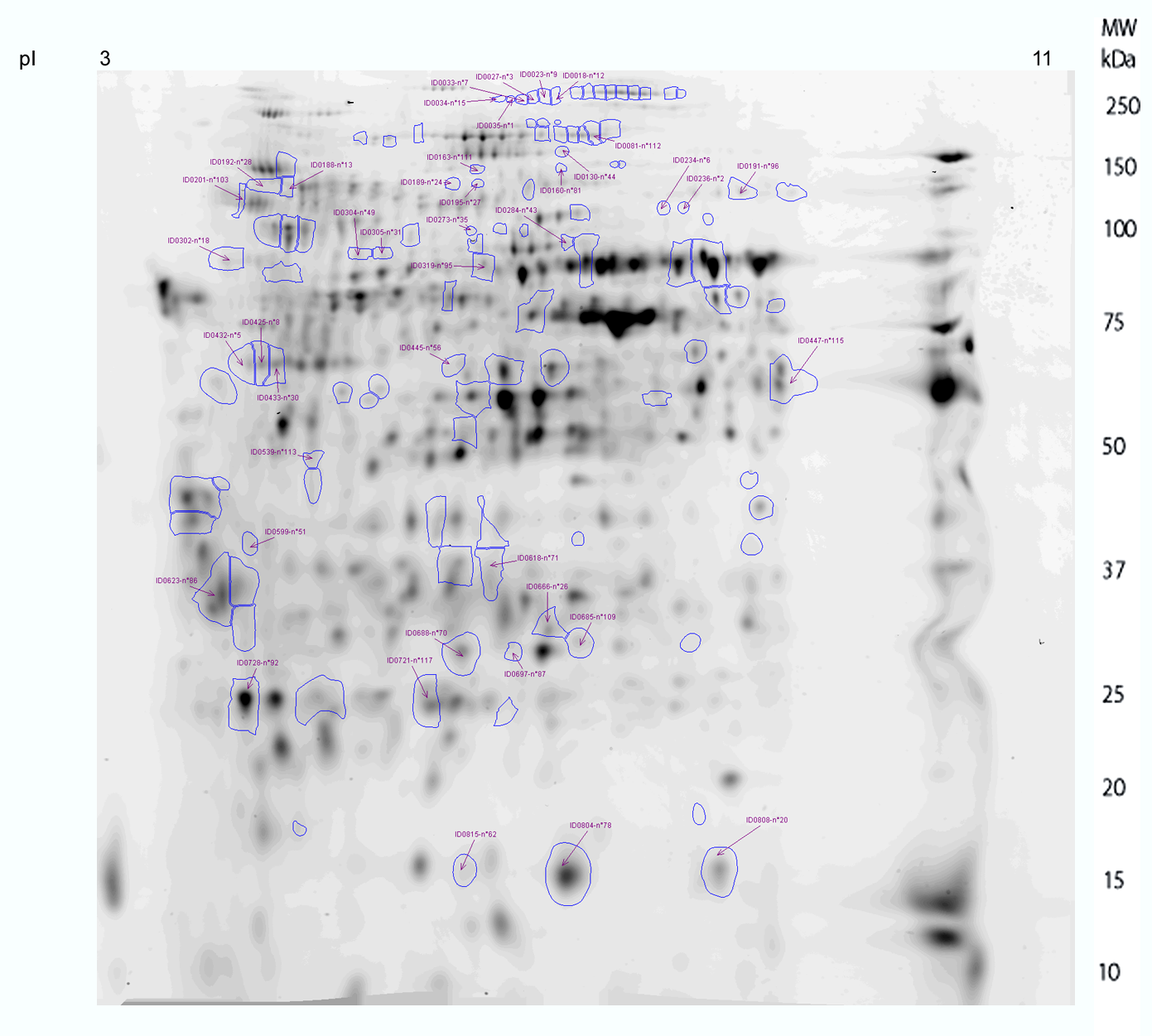

Supplement: Figure S4 — 2D-DIGE synthetic gel of Ae. aegypti midgut extracts showing spots modulated after analysis of CHIKV/DENV-2 profiles. Identification numbers (ID) and the range of each spot is shown on the gel. (5.24 MB TIF) [file pone.0013149.s008.tif]
